# Most users do not follow political elites on Twitter; those who do show overwhelming preferences for ideological congruity

**DOI:** 10.1126/sciadv.abn9418

**Published:** 2022-09-30

**Authors:** Magdalena Wojcieszak, Andreu Casas, Xudong Yu, Jonathan Nagler, Joshua A. Tucker

**Affiliations:** ^1^ University of California, Davis, Davis, CA, USA.; ^2^ University of Amsterdam, Amsterdam, Netherlands.; ^3^ Vrije Universiteit Amsterdam, Amsterdam, Netherlands.; ^4^ Center for Social Media and Politics, New York University, New York, NY, USA.

## Abstract

We offer comprehensive evidence of preferences for ideological congruity when people engage with politicians, pundits, and news organizations on social media. Using 4 years of data (2016–2019) from a random sample of 1.5 million Twitter users, we examine three behaviors studied separately to date: (i) following of in-group versus out-group elites, (ii) sharing in-group versus out-group information (retweeting), and (iii) commenting on the shared information (quote tweeting). We find that the majority of users (60%) do not follow any political elites. Those who do follow in-group elite accounts at much higher rates than out-group accounts (90 versus 10%), share information from in-group elites 13 times more frequently than from out-group elites, and often add negative comments to the shared out-group information. Conservatives are twice as likely as liberals to share in-group versus out-group content. These patterns are robust, emerge across issues and political elites, and exist regardless of users’ ideological extremity.

## Teaser

Few users follow politicians, pundits, and news media on Twitter; those who do strongly prefer ideological congruity.

## INTRODUCTION

Social media platforms are the primary source of political information for a growing number of citizens (*1*, *2*). This profoundly shifts the ways users encounter information and places individuals in unique media environments characterized by information flows curated by the users themselves and filtered through their social contacts. Although these changes facilitate exposure to different perspectives and connections with diverse people, they may also lead to the emergence of insular online communities where users follow members of their political group and share information consistent with their views (*3*, *4*). Such insular communication is feared to fuel extremity, exacerbate interparty hostility, and ultimately thwart consensual governance (*5*–*8*).

Given their democratic consequences, burgeoning research aims to describe such political congruity—which we refer to concisely as political biases in this manuscript—in users’ behaviors on social media (*9*). Existing evidence regarding the prevalence of these political biases, however, is inconclusive. Some studies show that bloggers primarily connect to sources from their ideological in-group (*10*, *11*) and that social media users exchange political information with copartisans (*12*), disproportionately follow politicians (*13*) and other users (*14*) from within their ideological group, and tend to share messages from their own party rather than those from other parties (*15*). Yet, others suggest that opposing partisans largely follow the same political accounts (*16*) and discuss nonpolitical topics across party lines (*12*). The extent of ideological asymmetries in these behaviors is also unclear: Sometimes, liberals are found to engage in more like-minded following than conservatives (*16*, *17*), and other times, they are shown to share more information across party lines (*12*, *18*), and yet other studies show that these asymmetries depend on the behaviors examined (*14*). This mixed evidence may be due to distinct samples and different behaviors studied in past work, such as who users follow (*16*) versus whether they retweet information about a few selected policies (*12*, *18*). A comprehensive approach that integrates these distinct behaviors in one analysis on a large random sample is lacking.

Here, we advance past work on political biases in users’ online behaviors in three key ways. First, we focus on users’ engagement with information produced by political elites, arguably the most influential and politically active users, testing whether these engagements are motivated by political bias. Unlike ordinary users, politicians, pundits, and news media contribute the overwhelming majority of political content and dominate online discussions (*19*). On social media platforms, politicians engage with constituents, “broadcast” information about their activities, and shape the political agenda (*19*). In turn, journalists and news media increase the reach of their stories and play a central role in the content that gets shared on social media platforms (*20*). The online activities of journalists, pundits, and news outlets are especially important as many citizens receive news through social network sites, not from producers directly (*21*).

Elite communication is also central to attitudes and behaviors of the electorate and may exacerbate unprecedented partisan conflicts in America (*5*). Elite cues can distort citizens’ policy preferences (*22*–*24*) and—by making interparty divisions clearer—polarize their attitudes (*22*, *25*). Elite communication makes people’s partisan identities more salient and casts politics as us-versus-them conflict, intensifying out-group hostility (*26*). Therefore, accounting for whether and how users engage with politicians, pundits, and news organizations on social media has clear societal implications.

Second, we integrate three distinct behaviors studied separately in past work and, in doing so, offer a more complete portrayal of political biases in users’ behavior on social media. We examine (i) the following of in-group versus out-group political elites, (ii) the sharing of their messages (i.e., retweeting), and (iii) adding comments to the shared messages of in-group versus out-group elites (i.e., quote tweets). These three behaviors reflect distinct affordances of social media platforms and have different implications for users themselves and online discourse at large. Following—although important—does not guarantee exposure to and interactions with the elite accounts that one follows. It is also rather passive and “private” in nature (apart from mere exposure, which we cannot examine). In turn, sharing and commenting represent more active engagements with elite messages, are more public in nature, and hence have a greater impact on the online public sphere. Diffusion through sharing increases information reach and shows how messages spread on social media (*16*). Also, sharing often indicates trust in the message and its source, agreement with the message (*27*), and users’ motivation to strategically construct and present their political identity to their online networks (*21*).

Sharing or reteweeting information, moreover, affords users an underanalyzed ability of adding comments to the shared messages. Theoretical frameworks of social identity establish that once individuals identify with a group, they aim to maintain and enhance their identity by positively distinguishing themselves and their in-group from the out-group (*28*–*30*). In the context of partisan communication on social media, this need for distinctiveness, or a unique positive social identity, can be met by sharing in-group content with positive comments. However, another way to enhance in-group status and achieve the need for distinctiveness is to attack or derogate the out-group (*31*). This phenomenon is visible in partisan media, which do cover the out-party, but often in derogatory ways, referring to it as Nazis or communists (*32*–*34*).

This phenomenon, although consequential, has been largely overlooked in the online environment. In the context studied, users may well retweet messages from across the political aisle, but do so to criticize the message or its source. Assessing the sentiment of the comments added to the shared elite information can show whether the assumed “endorsement by sharing” is undercut by mocking or criticism. If it is (e.g., conservatives retweeting Biden only to mock him), encountering information from out-group elites could actually have the effect of reinforcing political biases online.

Our third core contribution lies in putting in perspective extant concerns about political biases in online communication by examining a large random sample of Twitter users that contains both politically engaged and politically disengaged users. Most past work a priori explores online behaviors among politically engaged citizens, such as those who use political hashtags (*10*, *11*, *18*, *35*, *36*). However, those politically active users are far from being representative of social media users at large (*37*). Those citizens are more strongly partisan (*38*) and hence driven by confirmation bias or the need to “be right” and protect their viewpoints and political identity (*39*). Accordingly, studies on politically engaged users typically find political biases in their online behaviors, namely, following and engaging with one’s partisan in-group. The impression that political biases on social media are prevalent is further reinforced by the fact that the highly partisan users discuss salient policies online substantially more than other groups of users (*19*). Furthermore, those who are ideologically and affectively polarized are the ones who also amplify highly polarizing and sometimes misinformative content online (*40*, *41*). As a result, partisan and politically active users increase the visibility of political and polarizing information among their less partisan and engaged friends and followers and send the signal to recommendation algorithms to further amplify this content on social media platforms (*42*, *43*). In short, extant focus on this small group portrays an important yet incomplete picture of allegedly prevalent political biases in online behaviors.

In contrast, a solid majority of social media users are unlikely to engage in such biased, partisan, and polarizing online behaviors. Growing evidence shows that news and politics constitute a small fraction of people’s information diet. This is the case on social media: News makes up only 4% of News Feed on Facebook (*44*), public affairs more broadly comprise 1.8% of the average News Feed of college students (*45*), and only about 1 in 300 outbound clicks from Facebook correspond to substantive news (*46*). This is also the case online more broadly: Only between 2% (*47*) and 7 to 9% (*48*) of all URLs visited by large samples of Americans are news domains, and across mobile and desktop, news comprises only 4.2% of total online consumption (*49*).

Hence, the aforementioned biases in online communication are likely to follow the power law distribution, in that the majority of users are likely to be politically disengaged and not follow politicians, pundits, and news media organizations, and the small group that does engage with political elites is likely to be vocal, visible, and politically biased. Such power law distribution is increasingly detected with regard to (problematic) political behaviors online. On social media, a small share of highly active users produce the vast majority of content [e.g., the most active 25% of U.S. Twitter users produce 97% of all tweets (*37*)], a small fraction of people share fake news online [e.g., 1% of Twitter users accounted for 80% of exposures to fake news sources, and 0.1% accounted for 80% of all fake news sources shared (*50*)], and small groups of extreme partisans generate a majority of views to and engagements with partisan media on platforms (*16*). Because we start with an incredibly large random sample, we can offer generalizable evidence on these power laws in engagement with politicians, pundits, and news media among diverse, politically inclined and not, ordinary Twitter users.

To examine whether users engage with in-group versus out-group political elites in ways that reinforce political biases, we rely on 4 years of data (2016–2019) from a random sample of about 1.5 million Twitter users. We study whether they follow more than 2500 American political elite accounts and also examine instances in which they shared or quoted tweets from these accounts, which include about 20 million retweets in total. After using a validated method for estimating the ideology of regular and elite accounts (*12*) and a convolutional neural network (CNN) for classifying the sentiment of the quotes (*51*), we use these data to address five progressively specific questions: (i) What is the proportion of users who follow political elites? Among those who do: (ii) What proportion follows in-group versus out-group elites? (iii) What is the proportion of in-group versus out-group elite information shared by users? (iv) What is the sentiment—positive, neutral, or negative—of the commentary added to the tweets they share from in-group versus out-group elites? (v) Are there ideological asymmetries in these online behaviors?

Our analyses yield three clear pictures. The first is of a political vacuum on Twitter. The majority of American Twitter users do not follow politicians, pundits, or news media (59.6% of our sample), and only 23% follow more than three political elites. Second, when focusing on this small group, the second picture that emerges is one of pronounced political biases in users’ behaviors vis-a-vis political and media elites. In-group elites are followed at much higher rates than out-group accounts (around 90% versus 10%), and tweets from in-group elites are shared overwhelmingly more frequently than out-group tweets (at about a 13:1 ratio). The sharing of out-group elite is extremely limited, accounting for 7% of the retweets in our sample. Moreover, the sentiment of the comments on out-group tweets works to introduce further bias to this cross-party sharing: Quote tweets (or the added commentary) not only constitute a much larger percentage of out-group than in-group shares (about ^1^/_3_ versus less than ^1^/_10_) but also are negative two-thirds of the time when accompanying out-group tweets. Across 20 million retweets of elite content, only 5% were of out-group elites without any negative commentary. Third, we find important ideological asymmetries: Conservative users are roughly twice as likely as liberals to share in-group versus out-group content, as well as to add negative commentary to out-group shares. These patterns hold when accounting for the proportion of in-group versus out-group elites followed (i.e., users share in-group elites not only because they follow them more), across elite actors (but especially for politicians) and numerous issues, ranging from the economy to civil rights.

## RESULTS

Materials and Methods provides additional details on the data and methodology, and the Supplementary Materials offer a detailed description of all materials and methods used within this study as well as additional robustness checks, extended discussion of the machine learning classifiers, and alternative classifications. For 4 years (2016–2019), we tracked the messaging activity of a random sample of 1,437,774 Twitter users as well as an extensive set of 2624 political elite accounts. In this project, we focus on national political elites because of their visibility and national-level influence on public opinion and the political process. This focus is also in line with recent findings, which suggest that American political behavior is increasingly more nationalized, with voters being more engaged with and knowledgeable about national- than local-level issues and politics (*52*).

Our basic descriptives offer an important corrective to extant concerns about political biases on social media. We find that only 40.4% of the users (580,921 of 1,437,774) follow one of these elites or more. The remaining 59.6% follow no political actors whatsoever, although the list includes key politicians (e.g., Donald Trump and Joe Biden), prominent pundits (e.g., Rachel Madow and Sean Hannity), and the most popular media outlets (e.g., MSNBC and Fox News) (see section S10 for the full distribution of how many elite accounts users follow). Only 23% of users follow three or more elite accounts. Overall, the majority of American Twitter users are not sufficiently interested in politics to follow even a single political or media elite from our list (RQ1). To put this finding in perspective, we compare the following of political elites to that of celebrities related to music (e.g., Katy Perry), sports (e.g., Lebron James), TV-film (Oprah Winfrey, Kim Kardashian, and Tom Hanks), literature (Paulo Coelho), and fashion (Kendall Jenner), among other popular celebrities. We rely on a publicly available list of top 1000 Celebrity Twitter accounts (https://gist.github.com/mbejda/9c3353780270e7298763). We find that 70.7% of the users in our full sample follow at least one of the celebrities in the list, and more than 53.2% follow at least three of them, a stark difference compared to how many follow political elites. On average, the full set of users that we study follow about 10.7 celebrities but only 3.35 of the journalists, 1.52 of the politicians, and 1.13 of the media accounts on our list. Further details on these differential patterns are shown in section S12. To examine whether the politically engaged users follow in-group versus out-group elite accounts (RQ2), we first use the Bayesian spatial following model estimated by Barbera *et al.* (*12*) to assign ideological scores, on the same continuous scale, to political elites and ordinary Twitter users. We obtained an ideology score for 180,203 users. Although this group of politically engaged users is small relative to our total random sample (13% of 1,437,774), it accounts for 86% of all the shares of elite accounts sent by all ordinary users in our sample during the 4 years analyzed and is also politically consequential, as we note in Discussion.

**Fig. 1.**
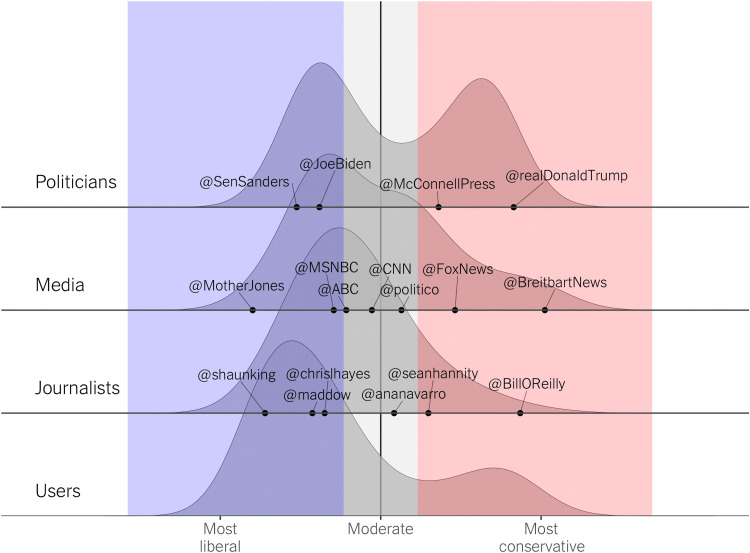
Ideology estimates. Distribution of the estimated ideology of the actors and ordinary users under study.

We then split the continuous ideology scale into a liberal, moderate, and conservative space and classify the accounts accordingly. Users with a score lower than 0 were classified as liberal; between 0 and 1.2, moderate; and higher than 1.2, conservative. Figure 1 shows the distribution of the ideology and the face validity of our measure. We focus on liberal and conservative elites and ordinary users (i.e., the blue and red areas in Fig. 1), excluding moderates, for whom a clear political in-group and out-group cannot be determined (keeping 1721 elite accounts and 151,063 ordinary users). Section S13 shows that our findings hold when including and classifying moderates into liberals and conservatives (using the vertical line in Fig. 1 as a cutoff point). In total, we examine 407 politicians (193 liberal and 214 conservative; 82 moderate accounts were excluded), 1234 pundits (969 liberal and 265 conservative; 782 moderate accounts were excluded), and 78 media organizations (51 liberal and 27 conservative; 39 moderate accounts were excluded). We also examine 115,589 liberal users and 35,474 conservative users (excluding 29,140 moderate ones from the 180,203 for which we obtained ideology scores).

**Fig. 2.**
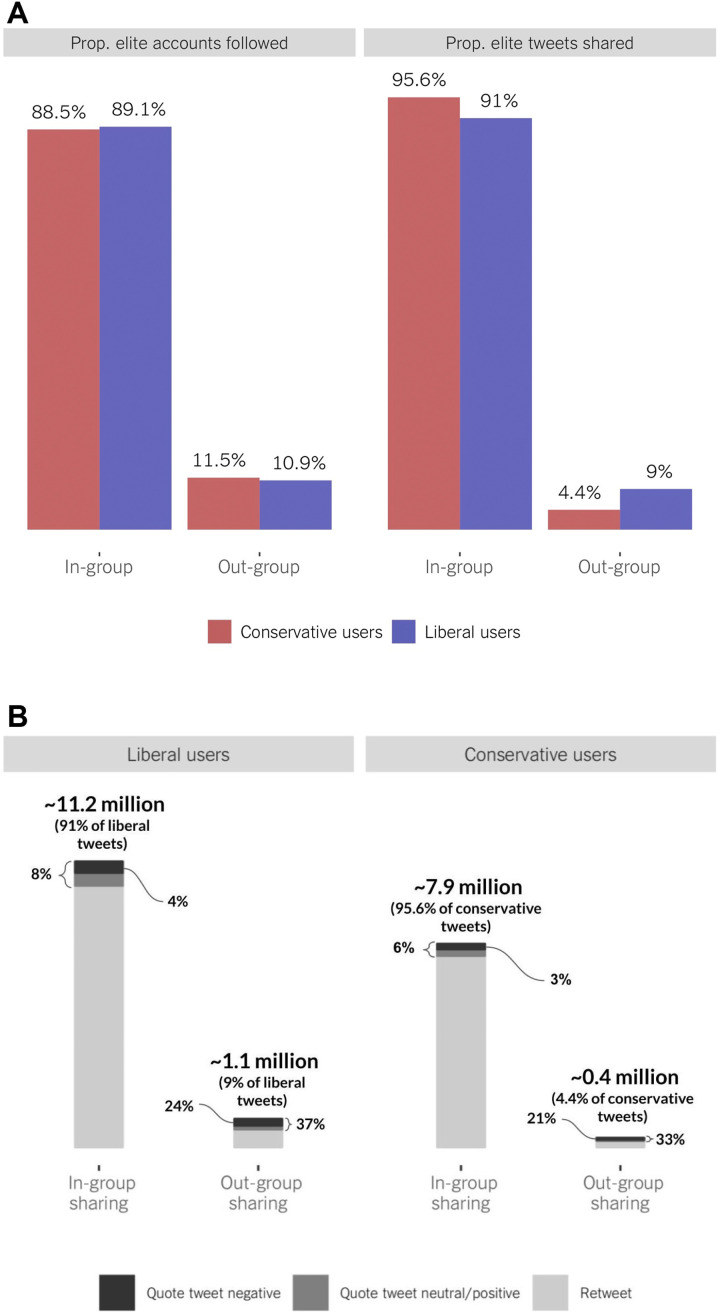
Amount of in-group (versus out-group) following and sharing. (**A**) Proportion (of all elite accounts that users follow) that are in-group versus out-group elite accounts and proportion (of all elite tweets that users shared) that are from in-group versus out-group elites (retweets and quote tweets pooled together). (**B**) How often ordinary users share (retweet versus quote tweet) messages from in-group versus out-group elites [providing further details about the right panel in (A)].

Pronounced differences depicted in the left panel of Fig. 2A reveal clear biases among politically active users: In-group politicians, pundits, and media are followed at much higher rates than out-group political elites (around 90 versus 10%). We do not observe any major difference between conservative (88.5% in-group versus 11.5% out-group elites followed) and liberal (89.5% in-group versus 10.9% out-group) users.

Because, as aforementioned, following is rather private, we take the next step, asking whether users actively share content from in-group versus out-group elites (RQ3). We collect all tweets quoting (i.e., retweets with a comment) or retweeting (i.e., tweets shared without any commentary) messages sent by elite accounts, 20,731,455 message shares in total. We find strong evidence that it does. Of all elite tweets that users share (with or without commentary), about 93% are from elites consistent with the users’ ideology. In general, for every out-group tweet a user shares, the user shares around 13 tweets from the in-group elite. Comparing Fig. 2A to Fig. 2B suggests that this bias is greater for sharing than following: Conditional on the number of in/out-group elite accounts followed, users have yet higher propensity to share in-group messages. The pattern is consistent across elite actors, although users are more likely to share tweets from in-group versus out-group media and pundits than politicians (see section S5). These biases are not a function of a few extreme users sharing information from few extreme elite sources: The levels of in-group sharing are also very high (although not as high) among users with low ideological extremity (see section S7).

We find clear ideological asymmetries. Although in-group elite content represents a similar proportion of all elite sharing by conservative (95.6%) and liberal (91%) users, the ratio of in-group to out-group shares is markedly different: 10:1 for liberals (11.2 million in-group/1.1 million out-group shares) and 20:1 for conservatives (7.9 million in-group/0.4 million out-group). As Fig. 2A shows, this cannot be explained by conservative users following fewer out-group elites.

The extent and nature of political biases further depend on the commentary added to the shared elite messages. Do users add negative comments when retweeting out-group messages (RQ4)? To establish the sentiment of the comments, we manually annotated a random sample of quotes for whether they were negative, neutral, or positive toward the original tweet (see Materials and Methods for the details on the annotation and section S8 for examples of labeled quote tweets). We used those annotated data to train a CNN to predict the tone of the remaining quotes (see section S8). We emphasize that the findings are robust to using sentiment predictions generated by a support vector machine and an Ensemble of several ngram-based models (see section S3).

Two patterns emerge. First, users add comments at a higher rate when sharing out-group tweets (37% for liberals; 33% for conservatives) than when sharing in-group messages (8 and 6%, respectively), suggesting that many users retweet out-group elites to express their stance, rather than endorse uncritically (see Fig. 2B). Second, the machine learning sentiment predictions find that, relative to all messages shared, users are more likely to add a negative comment to an out-group tweet (24% of all out-group shares for liberals; 21% for conservatives) than to an in-group tweet (4% of all in-group shares for liberals; 3% for conservatives). This translates into users adding negative commentary to an out-group elite tweet six times (liberals) and seven times (conservatives) more often than to an in-group tweet. In short, on the rare occasions that users share tweets from across the aisle, they do so to promote the in-group perspective: 63% of all quote tweets from out-group elites are shared with negative annotation.

**Fig. 3.**
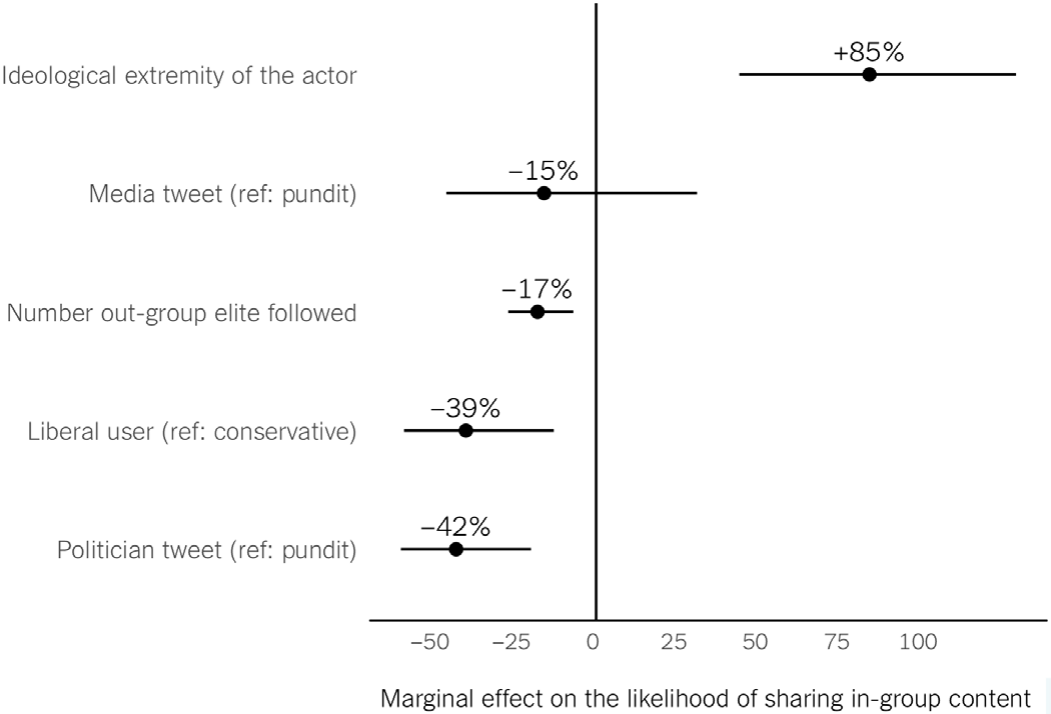
Logistic regression predicting whether users share in-group versus out-group elite tweets. Marginal effects, on in-group sharing, of the elite account being a politician or media (versus pundit), the ideological extremity of the elite actor, the ideology of the users, and the number of out-group elite accounts that a user follows. For continuous variables (ideological extremity and number of out-group elites followed), we report the marginal effect of 1 SD change.

We now offer more stringent evidence on these biases in engagement and on ideological differences by accounting for potential confounders (RQ5). First, we estimate a logistic regression model predicting the likelihood that users share in-group versus out-group elite tweets as a function of the ideology of the user (liberal versus conservative), the type of the elite (politicians, pundits, and news media), and the ideological extremity of the elite actor (i.e., folded continuous ideology score). To minimize the threat that our findings are driven by some users following many and others following few or no out-group elites, we control for the number of out-group accounts followed by each user (see section S1 for point estimates and 95% confidence intervals). As Fig. 3 shows, compared to conservative users, liberals are 39% less likely to share messages from in-group elites, even after controlling for these factors. We also find lower in-group sharing rates for politicians than pundits, indicating that tweets by out-group politicians are more likely to be shared (likely to be criticized, as we find above). Also, the more extreme the political elite actor, the more likely their tweets are shared by in-group users, suggesting the appeal of polarizing content and elites.

**Fig. 4.**
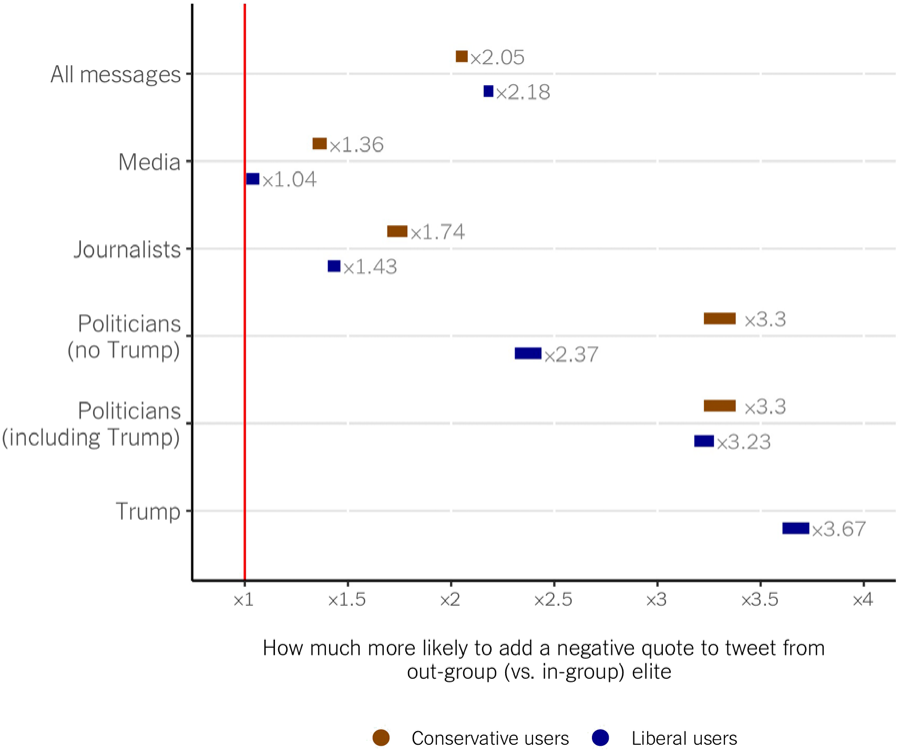
Six multinomial models predicting the likelihood of attaching negative comments when sharing tweets from out-group (versus in-group) elite accounts. Marginal effects for the elite tweet being from an out-group (rather than in-group) account.

Second, to better understand the conditions under which users negatively comment on out-group messages and to explore partisan differences, we estimate a set of ordinal logistic regression models predicting the sentiment of the comments on the shared tweets as a function of whether the retweet was from an in-group versus out-group elite and all the covariates from the model above. Figure 4 reports the results of six models (see section S2 for the coefficient tables). Across all messages (top row), a tweet of an out-group versus in-group elite is substantially more likely to be shared with a negative versus a neutral or positive comment, even after accounting for potential confounders. These patterns emerge among liberals and conservatives alike (2.18 times more likely for liberals and 2.05 for conservatives). Crucially, elite type and the ideology of ordinary users matter (rows below). Tweets from out-group politicians are most likely to be shared with a negative commentary, followed by out-group pundits and media. Despite the aggregate similarity, conservatives are more likely to add a negative comment to retweets from out-group politicians (3.3 times more likely versus 2.37), journalists (1.74 versus 1.43), and especially out-partisan media (1.36; liberals are only 4% more likely to negatively comment on out-group than on in-group media retweets). Quote tweets of Donald Trump are the reason for the aggregate similarity between liberals and conservatives. These tweets represent a large portion of the quote tweets in our data (around 20%), and liberal users overwhelmingly share them with negative comments. These models are robust to sentiment predictions from alternative machine learning models (see section S3). Overall, although both groups are similarly biased in their following patterns, conservative users exhibit greater bias by sharing messages from in-group elites and also—except for Trump for liberals—have a higher propensity to negatively comment on the out-group messages they share.

**Fig. 5.**
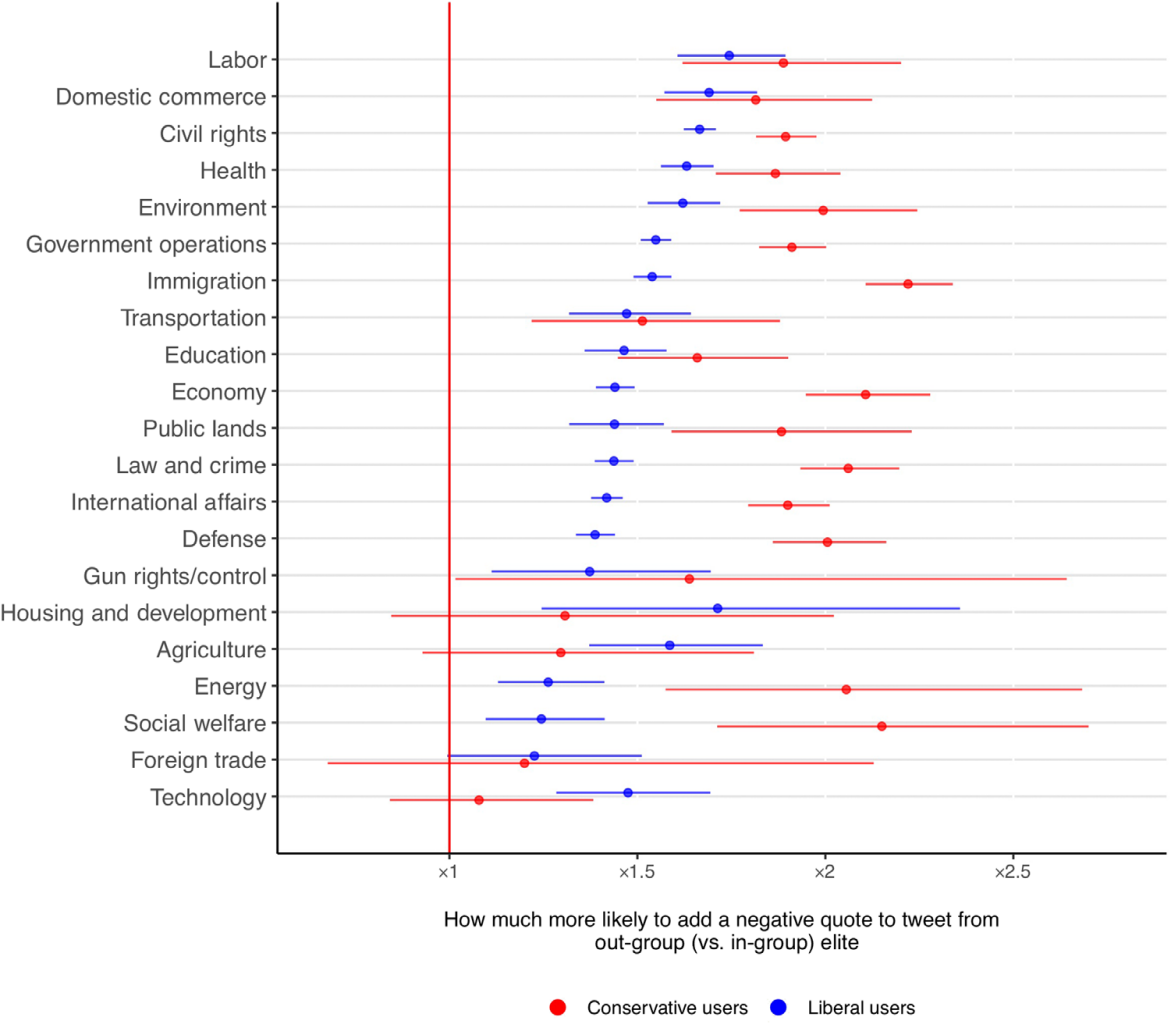
Twenty-one multinomial models predicting the likelihood of attaching negative comments when sharing tweets from out-group (versus in-group) elite accounts. For each of the policy areas discussed in the original elite tweets, we estimate the marginal effect of the tweet being from an out-group (rather than in-group) account.

Last, to assess whether these patterns depend on specific topics (e.g., when elites discuss hot-button issues versus complex policies), we trained a CNN to predict the presence of topics from the Comparative Agendas Project in the tweets (see section S9) and estimated multinomial models predicting the sentiment of the commentary on quote tweets about each topic, independently of the type of the elite. The political biases detected in our analyses are reinforced with negative commentary on divisive issues in American politics, such as immigration or civil rights, more than on the technical ones, such as technology or foreign trade. Yet, with a couple of isolated exceptions, regardless of the policy discussed, users from both ideologies are always more likely to add a negative comment to out-group rather than in-group messages (see Fig. 5).

## DISCUSSION

Our findings offer comprehensive evidence on political biases in people’s engagement with politicians, pundits, and news organizations on social media. Given the different affordances of Twitter and the potentially distinct effects that various user behaviors have on the users themselves, their social networks, and the online public discourse, we attend to the following and the sharing of in-group versus out-group elites as well as to the addition of positive or negative commentary to the shares. We asked whether a large random sample of users engages with in-group (rather than out-group) politicians, pundits, and news media on Twitter in ways that reinforce and exacerbate the feared insular communication patterns online. We offer three big takeaways.

First, most Twitter users do not follow or engage with any political elites online. This demonstrates a dichotomy between elite use of Twitter—politicians, pundits, and media (and also academics)—and mass use of Twitter. The elite discussion on the platform is important, but it is not necessarily observed directly by the masses. Given that Twitter users are more politically engaged than the general population to begin with (*37*), this finding of very low political elite following is unexpected. In our case, 59.6% of a random sample of users (856,853 of 1,437,774) were insufficiently politically interested to follow the accounts of the president, key senators, or major news media organizations. This bleak finding adds to some other evidence that many Twitter users do not follow news media (*16*) or members of Congress (*13*). It also aligns with the aforementioned work showing low absolute levels of news consumption online (*47*, *48*) and on social media more specifically (*44*), which users use primarily for entertainment (*37*). In our data, the following of celebrities is greater than that of any politician, pundit, or a news media organization: 70.7% of users follow at least one celebrity (athlete, musician, actor, etc.) compared to the 40.6% that follow at least one politically relevant elite account (see section S12).

Second, those who engage with political elites do so in an overwhelmingly one-sided way, displaying clear political biases in their behaviors. Users disproportionately follow and disseminate messages by like-minded politicians, pundits, and news media, rarely following and yet more rarely sharing cross-cutting elites. We counter the hope that these biases are confined to a small group of extreme users: Our patterns are robust (albeit naturally less pronounced) when examining all the users in our sample, including those ideologically moderate (see section S13), and are not driven by a few extreme users (see section S7). In addition, users not only are more likely to add a commentary to the out-group content they (rarely) share (i.e., quote tweets) but also add negative commentary to these shares. The negative sentiment of the commentary added to out-group retweets works to reinforce the ideological bubble. In summary, across the approximately 20 million shares of elite content that we analyzed, only 5% were of out-group elites without any negative commentary. Analyzing following, sharing, and commenting—an understudied feature of social media platforms—is one of the ways that this project extends past work and shows that Twitter users do exhibit strong and previously unaddressed political biases when engaging with politicians, pundits, and news media on Twitter.

Although some work suggests that people’s information diets partly overlap, online (*48*) and on social media (*16*), the evidence presented above suggests that there is little overlap between conservatives and liberals when interacting with political elites on Twitter. To speak more directly to the past studies, we checked this overlap in the following, sharing, and annotating of elite accounts (see section S11). Conservatives and liberals rarely follow elite accounts of the opposing ideology, although both groups jointly follow moderate elite accounts to some extent (about 35% of all elite accounts followed by liberal users are moderate accounts, and about 20% for conservatives), denoting some overlap. This overlap is smaller for users with a more extreme ideology, consistent with the idea that it is precisely the strong partisans who are most likely to engage in the most politically biased behaviors on social media. In short, the overlap between conservatives and liberals is mostly confined to moderate users following moderate politicians, pundits, and news media. Tellingly, these overlaps gradually decrease the more “public” the analyzed behavior becomes. That is, compared to the amount of moderate elite accounts that liberals and conservatives follow, retweets of moderate accounts represent a smaller proportion of their elite shares (about 30% for liberals and 6% for conservatives). In addition, although liberals are more likely to add a positive or neutral comment to tweets from moderate elite accounts, conservatives are more likely to add a negative commentary, emphasizing an even smaller overlap when it comes to commenting on tweets from moderate accounts.

Given these patterns of increasing political biases (and decreasing overlaps) in more public behaviors on social media, we speculate that these biases are partly due to the perceived polarization of one’s peer group. If citizens see others—be it the general public, their social network, or the imagined audiences of their tweets—as more polarized than they actually are (*53*), users may never share content from the other side, even if they are sympathetic to that content. In other words, social pressure to conform to (perceived) dominant group opinions may lead citizens to engage in “performative” sharing and commenting, which may explain and further exacerbate the detected biases. Future work should systematically test this idea and attend to disassociations between reading, following, sharing, and commenting, as each behavior entails different costs, sends different signals to one’s network, is subject to different pressures, and, hence, generates distinct political biases.

The third key finding regards ideological asymmetries, a key area for this research (*54*). Both conservative and liberal users are much more likely to follow in-group versus out-group elites, and both groups do so at similar rates. Also, although both groups are disproportionately more likely to retweet in-group than out-group elites, conservatives engage in cross-ideological diffusion substantially less. Also, apart from tweets from Donald Trump, conservatives tend to annotate out-group tweets with negative commentary more often than liberals do.

These asymmetries, consistent with prior research on political biases in users’ behaviors on social media (*12*, *55*, *18*), can be due to two interrelated factors. The work on distinct cognitive styles of political ideologues suggests that conservatives manifest cognitive styles such as dogmatism, rigidity, or uncertainty avoidance (*56*), which might predispose conservatives to shield away from and be more negative toward cross-cutting views [see (*12*, *57*)]. In addition, these asymmetries can be due to a broader social network ecosystem, such as the actions of friends and followers in right-leaning groups and also conservative users following and being targeted by more inauthentic accounts (*17*). Again, we encourage researchers to systematically attend to these differences and their underlying reasons.

These findings, although important, naturally do not offer a complete picture of political biases in all various information and communication behaviors on social media. For one, we do not examine interactions among ordinary users. Our theoretical and practical focus was on political elites, who dominate political discussions on platforms; have disproportionate influence on public, media, and policy agenda (*19*); and can further exacerbate or mitigate polarization. In addition, we do not analyze replies to elite tweets, focusing on retweeting and quote tweeting, behaviors that are more visible to one’s own followers than replies. Accounting for whether users replied to elite messages—and, if so, for the tone of the reply—would have offered a more complete portrayal of user engagement with elites on Twitter.

In a related vein, we cannot capture the content merely seen by the users in our sample, instead focusing on the more “active” behaviors of following, sharing (retweeting), and annotating (quote tweeting). Our focus may be underestimating the number of people exposed to elite messages. That is, some disengaged users may be exposed to the studied elites indirectly, through the retweets of their more engaged friends. Inasmuch as conservative/liberal users follow other conservative/liberal users [(*58*); see also (*17*)], this indirect exposure would be mostly to in-group and not out-group elites, thus introducing additional political biases on social media. This indirect exposure, moreover, could further exacerbate perceived or false polarization (*53*). The most partisan users are most likely to share political elites, and the extreme elites are most likely to be shared (*59*). As a result, apolitical users who do not themselves follow any elites would encounter content that is hyperpartisan. Furthermore, if out-group content is retweeted with an added commentary, those apolitical users would see messages that derogate the other side. This could create the perception that politics is divisive and polarized, further disengaging some citizens from the political process (*60*, *61*). Testing these indirect, inadvertent exposures to elite communications and analyzing their effects on the users are a worthwhile direction for future work.

We also encourage researchers to extend our work to a local level of Twitter discussions. In this project, we offered a foundational overview of political biases in following, sharing, and annotating on a large national scale, looking at the most powerful, and therefore potentially most frequently followed, political elites (*52*). Yet, some recent work suggests that contentious political issues are also discussed at the state level and that patterns of media use, political talk, and policy attitudes differ between localities within states (*62*, *63*). Future work should test whether similar biases in the following, retweeting, and quote tweeting of in-group versus out-group politicians and news media organizations emerge on local levels. We also hope that scholars will extend our approach to international contexts to examine whether the U.S. political ecosystem online is unique or whether Twitter users follow, share, and annotate in similar ways in multiparty and potentially less polarized systems.

Our findings have important implications for research and democracy at large. Despite the hopes that social media would reinvigorate American democracy by lowering information costs and access barriers and directly connecting representatives with their constituencies, most citizens do not engage with politicians, pundits, and news organizations on social media platforms. The unprecedented choice in the online environment reinforces the divide between the politically withdrawn and more politically active citizens. This latter group can now easily selectively engage with like-minded sources and information, disparage the out-group and its messages, and become yet more polarized.

When we witness a growing radicalization of certain groups in American society (and globally), decreasing support for democratic norms, and rising support for political violence (*64*), concerns about political biases in online behaviors are ever more pressing, no matter how small the groups engaging in those behaviors may be. Because these small groups are disproportionately more vocal, participatory, and used by mainstream news media to represent public opinion (*65*), they amplify the general public perception of ideological extremity, political biases, and unprecedented polarization. Yet, scholars and public observers need to keep in mind that these political biases are removed from the everyday information and communication ecosystem of most American citizens and that pulling these less engaged and more moderate citizens back into the democratic process may decrease political polarization online and offline.

## MATERIALS AND METHODS

### Data collection

The data were collected by the Center for Social Media and Politics at New York University in between 2016 and 2019. We generated a random sample of Twitter users in two ways. First, (i) before Twitter switched to 64-bit IDs in 2016, we automatically generated 32-bit random numbers and checked for whether they were existing Twitter users (first about 100,000 users). After the introduction of the longer Twitter IDs, (ii) we increased the size of the sample: We collected tweets mentioning a set of English stopwords (i.e., “the”) for numerous short amounts of time selected at random and then pulled the authors of those tweets and information about how frequently they tweeted, and we subsampled a set of authors with a tweeting distribution similar to that of the users in the list created using the first approach (i). For the second approach (ii), we selected times in the day when users from other English-speaking countries were the least likely to tweet to assure that the tracked users are in the United States. We do not impose other additional geographic restrictions, but given the U.S.-centric character of the English-speaking Twitter at the time we collected the data and the fact that we randomly sampled users messaging at times when users from other English-speaking countries are least likely to tweet, we expect most of these users to be located in the United States. Our findings hold when only looking at a set of 24,328 users we have confidently located in the United States (using the method described in SI B in itebarbera-who-2019) as shown in section S10.

In summary, we tracked the following, tweeting, retweeting, and quote tweeting activity of a random sample of 1,437,774 Twitter users for 4 years (2016–2019) by regularly pulling their timelines using the Twitter REST API. Specifically, we examined whether the users followed an extensive set of 2624 political elite accounts (489 politicians, 2016 pundits, and 119 media organizations), whether they have retweeted messages from these accounts, and whether they have used quote tweets to add comments to the retweeted messages. This full sample is used in our initial descriptive analyses addressing RQ1.

To address our subsequent RQs that focus on ideological in-groups and out-groups, we apply the ideology classification developed by Barbera *et al.* (*12*). We note that this classification has been extensively validated using external indicators on the aggregate and individual levels [figs. S2, S4, and S5 (*12*)]. We do not train a new model from scratch and use the model in (*12*) to generate an ideology score for the users and actors in our sample. Because the list of elite accounts used in (*12*) to generate an ideology estimation for ordinary users was slightly more restrictive than ours, there are some users who do not follow enough elites in the original list to estimate their ideology. Ultimately, as mentioned in the Results section, we were able to classify the ideology of 180,203 users in our sample.

In the analyses examining whether users actively share content from in-group versus out-group elites (RQ3), we include those users who retweeted or quote tweeted messages sent by the elite accounts on our list. In particular, we use 20,731,455 shares of elite accounts classified as liberal or conservative (moderates excluded) that were shared by 151,063 politically active users classified as liberal or conservative (moderates excluded as well). We included any quote tweet, even a quote tweet of someone that a user does not follow her/himself.

Last, to address RQ4 regarding the sentiment (positive, neutral, or negative) of the commentary added to the shared elite tweets (i.e., quote tweets), we needed to remove quote tweets too short for sentiment predictions (<5 words, after preprocessing the text). The final sample for these analyses is 1,469,708 tweets sent by 85,849 users (about 57% of the 151,063 politically active users classified as liberals or conservatives) quoting 1668 elites: 563,689 (38%) tweets quoting 402 politicians, 391,433 (27%) quoting 78 media organizations, and 514,586 (35%) quoting 1188 pundits. In general, 668,248 (46%) quoted conservative actors, and 801,460 (54%) quoted liberal actors.

### Classifiers

#### Sentiment classifier

To determine the sentiment of the commentary added to the shared elite messages, whether positive, neutral, or negative, we trained a CNN classifier predicting the sentiment of the quote tweets. First, we randomly sampled 8351 tweets from our full dataset of quote tweets. Four trained research assistants manually coded them for whether the quote was negative, neutral, or positive toward the message and/or the political actor, independently of the tone of the original message (Krippendorff’s alpha = 0.816). We used those annotated data to train the following five types of machine learning models predicting whether the commentaries were positive, neutral, or negative (multiclass models): (a) a Decision tree (TREE), (b) a K-neighbors model, (c) a support vector machine, (d) a majority-based ensemble model that took into account the output of the three previous ones, and (e) a four-layer CNN. For training (a), (b), (c), and (d), we transformed all text to lowercase, removed stopwords, and lemmatized the remaining tokens to lastly create a TF-IDF (term frequency-inverse document frequency) matrix that we used as model input. For the CNN model, we transformed all text to lowercase and used 300-dimension GloVe embeddings as inputs. We tested the accuracy of each algorithm using fivefold cross-validation and an 80/20 train-test split. As seen in section S8 and fig. S5, the CNN proved to be the most accurate of the five classifiers (see colored section S8 for additional details and validations). Given the superior performance, we use the CNN classifier to predict the tone of all quote tweets in our dataset. We also emphasize that the findings are robust to using sentiment predictions generated by a support vector machine and an Ensemble of several ngram-based models (see section S3).

#### Topic classifier

In addition, we automatically classified the topic of the content of the original tweets from elite accounts that we study. Given the large number of tweets, manual coding was not practical for the full corpus. To reliably and at-scale predict the topic of the original elite tweets, we trained a CNN predicting whether each tweet discussed 1 of the 20 topics of the Comparative Agendas Project (*66*). In section S9, we provide detailed information about the model architecture, how it was trained, and its performance.

## Supplementary Materials

### This PDF file includes:

Sections S1 to S13 Tables S1 to S16 Figs. S1 to S11 References
