# Supplementary material for: Most users do not follow political elites on Twitter; those who do show overwhelming preferences for ideological congruity
Source: Sci Adv. 2022 Sep 30;8(39):eabn9418. doi: 10.1126/sciadv.abn9418 (PMC9524832; doi:10.1126/sciadv.abn9418)
Supplement: Supplementary file 1 — Sections S1 to S13 Tables S1 to S16 Figs. S1 to S11 References [file sciadv.abn9418_sm.pdf]

Science Advances
AAAS

Supplementary Materials for

Most users do not follow political elites on Twitter; those who do show overwhelming preferences for ideological congruity

Magdalena Wojcieszak *et al.*

Corresponding author: Magdalena Wojcieszak, mwojcieszak@ucdavis.edu

Sci. Adv. **8**, eabn9418 (2022)
DOI: 10.1126/sciadv.abn9418

This PDF file includes:

Sections S1 to S13
Tables S1 to S16
Figs. S1 to S11
References

1 Logistic regression model predicting the likelihood that users share in-group versus out-group elite tweets

Table S1: Logistic regression model predicting the likelihood that users share in-group versus out-group elite tweets

	pe	lwr	upr
Ideological extremity of the actor	.84	.45	1.34
Media tweet [Ref: Pundit]	-.15	-.46	.33
Number out-group elite followed	-.16	-.25	-.06
Liberal user [Ref: Conservative]	-.39	-.59	-.13
Politician tweet [Ref: Pundit]	-.42	-.59	-.19

Note: pe = point estimates. lwr = lower bound of the 95% confidence interval. upr = upper bound of the 95% confidence interval.

2 Ordinal logistic regression models predicting the sentiment of the comments on the shared tweets

Table S2: Ordinal logistic regression models predicting the sentiment of the comments on the shared tweets (Liberal users)

Variable	All Messages	Media	Journalists	Politicians (No Trump)	Politicians (Incl. Trump)	Trump
Source (Out-group)	-.78* (.01)	-.03* (.02)	-.36* (.01)	-.85* (.01)	-1.18* (.01)	-1.31* (.01)
Num. Followers (actor)	.04* (.00)	.03* (.00)	-.03* (.00)	-.00 (.00)	.06* (.00)	-
Num. Friends (user)	-.01* (.00)	-.01 (.01)	-.02* (.01)	-.04* (.01)	-.01* (.00)	-.00 (.00)
Num. Followers (user)	.06* (.00)	.06* (.01)	.03* (.00)	.05* (.01)	.06* (.00)	.06* (.00)
Ideo. Extrimity (actor)	-.53* (.01)	-.05 (.03)	-.23* (.02)	-.58* (.04)	-.67* (.04)	-
Out. Elites Followed	.00* (.00)	.00 (.00)	.00* (.00)	.00* (.00)	.00 (.00)	-.00 (.00)
Negative | Neutral	.48* (.05)	.63* (.16)	-.36* (.13)	-.69* (.14)	.49* (.06)	-.15* (.07)
Neutral | Positive	.57* (.05)	.73* (.16)	-.27* (.13)	-.59* (.14)	.58* (.06)	-.06 (.07)
Controlling for Time	Yes	Yes	Yes	Yes	Yes	Yes
Controlling for Topic	Yes	Yes	Yes	Yes	Yes	Yes

Note: Number of actors' followers, number of users' friends, and number of a users' followers were log(x+1) transformed. Cell entries are regression coefficients with associated standard errors in parentheses. $*p < .05$

Table S3: Ordinal logistic regression models predicting the sentiment of the comments on the shared tweets (Conservative users)

Variable	All Messages	Media	Journalists	Politicians (No Trump)	Politicians (Incl. Trump)
Source (Out-group)	-.72* (.01)	-.31* (.01)	-.55* (.01)	-1.19* (.01)	-1.19* (.01)
Num. Followers (actor)	.02* (.00)	.02* (.00)	-.01* (.00)	.02* (.00)	.02* (.00)
Num. Friends (user)	-.03* (.00)	-.01* (.00)	-.02* (.00)	-.04* (.01)	-.04* (.01)
Num. Followers (user)	.04* (.00)	.04* (.00)	.03* (.00)	.06* (.01)	.06* (.01)
Ideo. Extrimity (actor)	.24* (.02)	.33* (.03)	.15* (.02)	.46* (.05)	.46* (.05)
Out. Elites Followed	.00* (.00)	.00* (.00)	.00* (.00)	.00* (.00)	.00* (.00)
Negative | Neutral	.31* (.06)	.57* (.08)	-.17 (.10)	.32* (.12)	.32* (.12)
Neutral | Positive	.43* (.06)	.70* (.08)	-.04 (.10)	.41* (.12)	.41* (.12)
Controlling for Time	Yes	Yes	Yes	Yes	Yes
Controlling for Topic	Yes	Yes	Yes	Yes	Yes

Note: Number of actors' followers, number of users' friends, and number of a users' followers were log(x+1) transformed. Cell entries are regression coefficients with associated standard errors in parentheses. $*p < .05$

3 Ordinal logistic regression models predicting the sentiment of the comments on the shared tweets using SVM and Ensemble

Table S4: Ordinal logistic regression models predicting the sentiment of the comments on the shared tweets using SVM (Liberal users)

Variable	All Messages	Media	Journalists	Politicians (No Trump)	Politicians (Incl. Trump)	Trump
Source (Out-group)	-.62* (.01)	-.05* (.02)	-.33* (.01)	-.75* (.01)	-.91* (.01)	-.99* (.01)
Num. Followers (actor)	.04* (.00)	.02* (.00)	-.01* (.00)	-.00 (.01)	.07* (.00)	-
Num. Friends (user)	-.00 (.00)	-.03* (.01)	-.01 (.01)	-.03* (.01)	.00 (.00)	.01* (.00)
Num. Followers (user)	.01* (.00)	.04* (.01)	.01 (.00)	.01 (.01)	.00 (.00)	-.00 (.00)
Ideo. Extrimity (actor)	-.45* (.01)	-.15* (.03)	-.16* (.02)	-.85* (.04)	-.88* (.04)	-
Out. Elites Followed	.00* (.00)	.00 (.00)	.00* (.00)	.00* (.00)	.00* (.00)	.00* (.00)
Negative | Neutral	-.42* (.05)	-.41* (.17)	-1.19* (.15)	-1.39* (.14)	-.33* (.07)	-1.11* (.07)
Neutral | Positive	-.38* (.05)	-.36* (.17)	-1.15* (.15)	-1.35* (.14)	-.29* (.07)	-1.07* (.07)
Controlling for Time	Yes	Yes	Yes	Yes	Yes	Yes
Controlling for Topic	Yes	Yes	Yes	Yes	Yes	Yes

Note: Number of actors' followers, number of users' friends, and number of a users' followers were log(x+1) transformed. Cell entries are regression coefficients with associated standard errors in parentheses. $*p < .05$

Table S5: Ordinal logistic regression models predicting the sentiment of the comments on the shared tweets using SVM (Conservative users)

Variable	All Messages	Media	Journalists	Politicians (No Trump)	Politicians (Incl. Trump)
Source (Out-group)	-.48* (.01)	-.23* (.01)	-.38* (.01)	-.77* (.01)	-.77* (.01)
Num. Followers (actor)	.02* (.00)	.03* (.00)	.00 (.00)	.02* (.00)	.02* (.00)
Num. Friends (user)	-.02* (.00)	-.00 (.01)	-.03* (.01)	-.03* (.01)	-.03* (.01)
Num. Followers (user)	.02* (.00)	.02* (.00)	.02* (.00)	.03* (.01)	.03* (.01)
Ideo. Extrimity (actor)	.26* (.02)	.32* (.03)	.13* (.03)	.45* (.05)	.45* (.05)
Out. Elites Followed	.00* (.00)	.00* (.00)	.00* (.00)	.00* (.00)	.00* (.00)
Negative | Neutral	-.56* (.06)	-.16 (.09)	-.79* (.11)	-.68* (.14)	-.68* (.14)
Neutral | Positive	-.51* (.06)	-.11 (.09)	-.74* (.11)	-.63* (.14)	-.63* (.14)
Controlling for Time	Yes	Yes	Yes	Yes	Yes
Controlling for Topic	Yes	Yes	Yes	Yes	Yes

Note: Number of actors' followers, number of users' friends, and number of a users' followers were log(x+1) transformed. Cell entries are regression coefficients with associated standard errors in parentheses. $^{*}p < .05$

Table S6: Ordinal logistic regression models predicting the sentiment of the comments on the shared tweets using Ensemble (Liberal users)

Variable	All Messages	Media	Journalists	Politicians (No Trump)	Politicians (Incl. Trump)	Trump
Source (Out-group)	-.62* (.01)	-.10* (.02)	-.33* (.01)	-.74* (.01)	-.91* (.01)	-.98* (.01)
Num. Followers (actor)	.04* (.00)	.02* (.00)	-.01 (.00)	.00 (.01)	.06* (.00)	-
Num. Friends (user)	-.00 (.00)	-.02 (.01)	-.01 (.01)	-.03* (.01)	.01 (.00)	.02* (.00)
Num. Followers (user)	.01* (.00)	.03* (.01)	.01 (.00)	.01 (.01)	.00 (.00)	-.00 (.00)
Ideo. Extrimity (actor)	-.41* (.01)	-.12* (.03)	-.11* (.02)	-.77* (.04)	-.79* (.04)	-
Out. Elites Followed	.00* (.00)	.00 (.00)	.00* (.00)	.00* (.00)	.00* (.00)	.00* (.00)
Negative | Neutral	-.41* (.05)	-.26 (.17)	-1.07* (.14)	-1.33* (.14)	-.35* (.06)	-.99* (.07)
Neutral | Positive	-.34* (.05)	-.19 (.17)	-1.00* (.14)	-1.27* (.14)	-.29* (.06)	-.92* (.07)
Controlling for Time	Yes	Yes	Yes	Yes	Yes	Yes
Controlling for Topic	Yes	Yes	Yes	Yes	Yes	Yes

Note: Number of actors' followers, number of users' friends, and number of users' followers were log(x+1) transformed. Cell entries are regression coefficients with associated standard errors in parentheses. $^{*}p < .05$

Table S7: Ordinal logistic regression models predicting the sentiment of the comments on the shared tweets using Ensemble (Conservative users)

Variable	All Messages	Media	Journalists	Politicians (No Trump)	Politicians (Incl. Trump)
Source (Out-group)	-.41* (.01)	-.20* (.01)	-.34* (.01)	-.67* (.01)	-.67* (.01)
Num. Followers (actor)	.02* (.00)	.03* (.00)	-.00 (.00)	.03* (.00)	.03* (.00)
Num. Friends (user)	-.02* (.00)	-.01 (.01)	-.02* (.01)	-.03* (.01)	-.03* (.01)
Num. Followers (user)	.02* (.00)	.02* (.00)	.01* (.00)	.02* (.01)	.02* (.01)
Ideo. Extrimity (actor)	.24* (.02)	.34* (.03)	.10* (.03)	.35* (.05)	.35* (.05)
Out. Elites Followed	.00* (.00)	.00 (.00)	.00* (.00)	.00* (.00)	.00* (.00)
Negative | Neutral	-.46* (.06)	-.10 (.09)	-.75* (.11)	-.59* (.14)	-.59* (.14)
Neutral | Positive	-.38* (.06)	-.02 (.09)	-.67* (.11)	-.52* (.14)	-.52* (.14)
Controlling for Time	Yes	Yes	Yes	Yes	Yes
Controlling for Topic	Yes	Yes	Yes	Yes	Yes

Note: Number of actors' followers, number of users' friends, and number of users' followers were log(x+1) transformed. Cell entries are regression coefficients with associated standard errors in parentheses. $*p < .05$

4 Ordinal logistic regression models predicting the sentiment of the comments on the shared tweets including moderates

Table S8: Ordinal logistic regression models predicting the sentiment of the comments on the shared tweets including moderates (Liberal users)

Variable	All Messages	Media	Journalists	Politicians (No Trump)	Politicians (Incl. Trump)	Trump
Source (Out-group)	-.58* (.00)	.01 (.01)	-.21* (.01)	-.72* (.01)	-1.06* (.01)	-1.21* (.01)
Num. Followers (actor)	.03* (.00)	.03* (.00)	-.02* (.00)	-.00 (.00)	.06* (.00)	-
Num. Friends (user)	-.02* (.00)	-.01 (.01)	-.02* (.00)	-.05* (.01)	-.02* (.00)	-.01 (.00)
Num. Followers (user)	.06* (.00)	.05* (.00)	.03* (.00)	.06* (.01)	.07* (.00)	.07* (.00)
Ideo. Extrimity (actor)	-.69* (.01)	.05* (.03)	-.27* (.02)	-.82* (.04)	-1.02* (.04)	-
Out. Elites Followed	.00* (.00)	.00 (.00)	.00* (.00)	.00 (.00)	-.00 (.00)	-.00* (.00)
Negative | Neutral	.14* (.04)	.76* (.11)	-.27* (.10)	-.80* (.12)	.23* (.06)	-.09 (.06)
Neutral | Positive	.23* (.04)	.86* (.11)	-.17 (.10)	-.71* (.12)	.33* (.06)	.01 (.06)
Controlling for Time	Yes	Yes	Yes	Yes	Yes	Yes
Controlling for Topic	Yes	Yes	Yes	Yes	Yes	Yes

Note: Number of actors' followers, number of users' friends, and number of users' followers were log(x+1) transformed. Cell entries are regression coefficients with associated standard errors in parentheses. $*p < .05$

Table S9: Ordinal logistic regression models predicting the sentiment of the comments on the shared tweets including moderates (Conservative users)

Variable	All Messages	Media	Journalists	Politicians (No Trump)	Politicians (Incl. Trump)
Source (Out-group)	-.51* (.01)	-.25* (.01)	-.36* (.01)	-1.09* (.01)	-1.09* (.01)
Num. Followers (actor)	.02* (.00)	.03* (.00)	-.02* (.00)	.02* (.00)	.02* (.00)
Num. Friends (user)	-.03* (.00)	-.00 (.00)	-.03* (.00)	-.04* (.01)	-.04* (.01)
Num. Followers (user)	.04* (.00)	.03* (.00)	.03* (.00)	.06* (.00)	.06* (.00)
Ideo. Extrimity (actor)	.17* (.01)	.18* (.02)	.18* (.01)	.17* (.04)	.17* (.04)
Out. Elites Followed	.00* (.00)	.00* (.00)	.00* (.00)	.00* (.00)	.00* (.00)
Negative | Neutral	.29* (.04)	.68* (.06)	-.07 (.07)	.01 (.10)	.01 (.10)
Neutral | Positive	.42* (.04)	.81* (.06)	.06 (.07)	.10 (.10)	.10 (.10)
Controlling for Time	Yes	Yes	Yes	Yes	Yes
Controlling for Topic	Yes	Yes	Yes	Yes	Yes

Note: Number of actors' followers, number of users' friends, and number of users' followers were log(x+1) transformed. Cell entries are regression coefficients with associated standard errors in parentheses. $*p < .05$

5 Ingroup and outgroup sharing patterns by elite type

In Figure 2 of the paper we show a large sharing echo chamber as it comes to messages from elite accounts: ordinary users are substantially more likely to share tweets from ingroup than outgroup elites. Additionally, we also show a substantive sentiment echo chamber: they are also more likely to add a negative commentary when quoting a tweet from an outgroup elite than an ingroup elite. In this SI section we show how these

pattern hold for the different types of elite groups we study (politicians, media, and journalists), as well as for elites of different ideology (liberal *v.* conservative).

In Figure S1 below we show that no matter to which actor type we look at, and from which ideology, ordinary users are more likely to share tweets from ingroup accounts. Descriptively, we see the difference between ingroup and outgroup sharing to be slightly more pronounced among liberals elites (so among conservative users), but the general ingroup-outgroup divide is common across elite type and ideology.

Figure S1: How often the tweets of different elite actors (and of different ideology) are shared by ingroup *v.* outgroup ordinary users

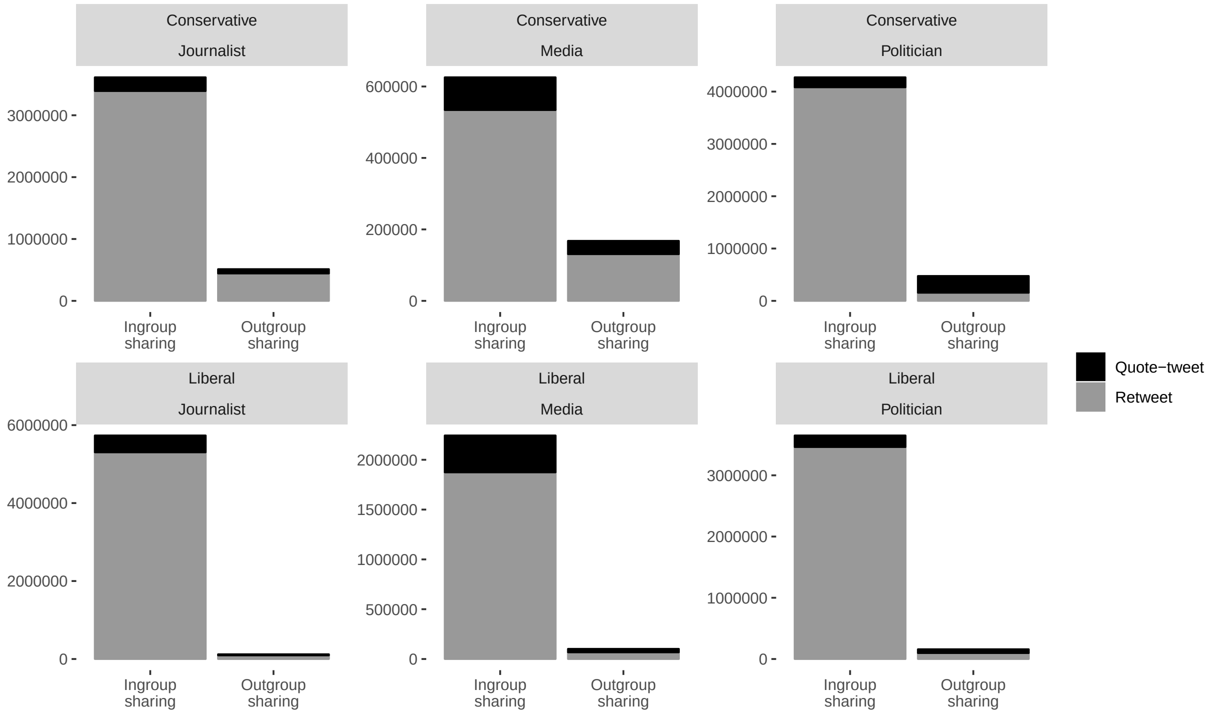

Then, in Figure S2 we zoom in on the quote tweets to show how often ordinary users decide to add a negative (or positive) commentary to tweets from the different types of elites. The general pattern is also consistent across elite type: users are more likely to add a negative comment when quoting tweets from outgroup rather than ingroup elites. Although the pattern is clearly more pronounced when looking at politicians rather than media organizations and pundits.

Figure S2: How often the tweets of different elite actors (and of different ideology) are shared by ingroup *v.* outgroup ordinary users

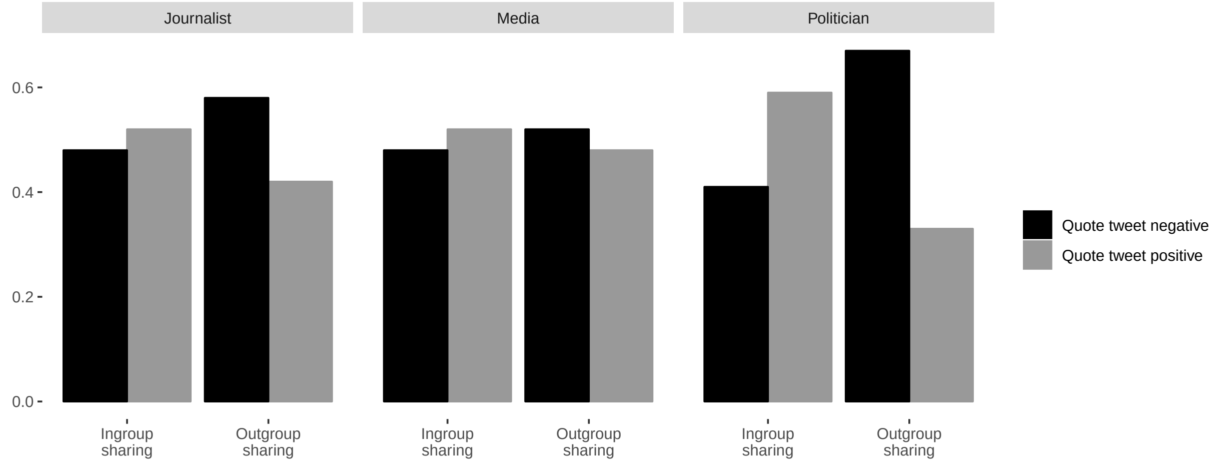

6 Ingroup sharing and ideological extremity of the actors

In the paper we show that the sample of Twitter users interested in U.S. politics we study overwhelmingly share ingroup rather than outgroup elite content from politicians, pundits, and the media (Figure 2). However, readers may be concerned that the high levels of ingroup sharing shown in the paper are driven by the users in our sample mostly engaging and sharing content from extreme elite accounts.

Figure S3: Ingroup sharing of tweets from elite accounts as a function of their ideological extremity.

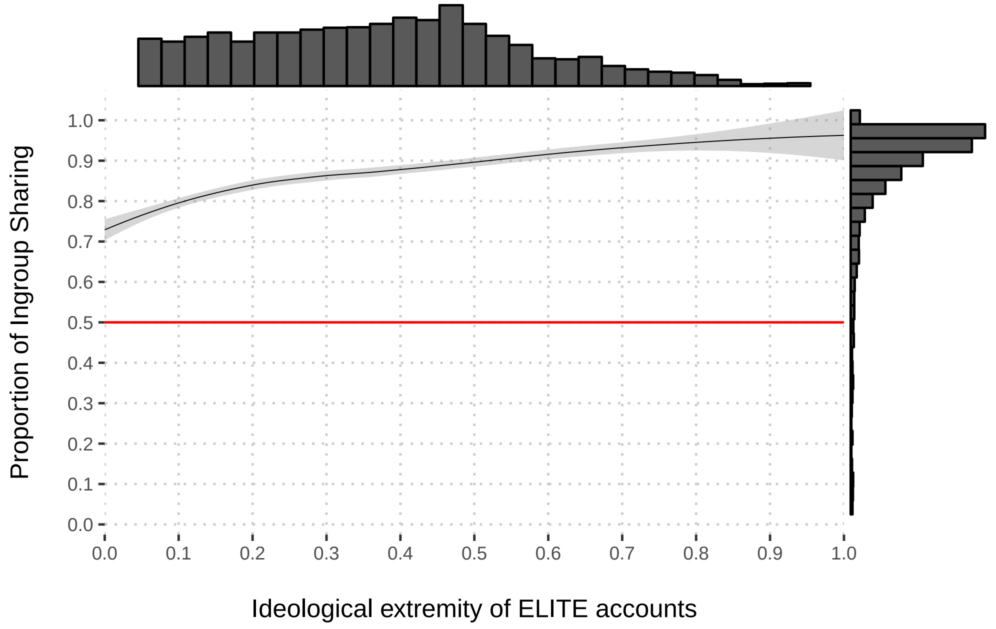

In Figure S3 we show how often tweets from elite accounts that we classified as liberal or conservative are shared by users of the same ideological liberal-conservative group (proportion of ingroup sharing). We folded and standardized (between 0 and 1) the ideology scores of the elite accounts in order to create an ideological extremity measure. In this new measure, very moderate liberal and conservative elite accounts have a value close to 0, and very extreme liberal and conservative elite accounts have values close to 1. The figure shows that most elite accounts have a relatively moderate ideological extremity score between 0 and 0.5 (see histogram at the top of the figure). We also observe that ingroup sharing is already very high among these moderate liberal and conservative elite accounts. On average, ingroup users are responsible for at least 70% of the shares of tweets from these accounts. So although the proportion of ingroup sharing is even higher for elite accounts with a very high ideological extremity score, overall we see high levels of ingroup sharing across the board. These results indicate that ingroup sharing is an extended pattern no matter the extremity of the source.

7 Ingroup sharing and ideological extremity of the users

Similarly to what we voiced in the previous section, readers may also be concerned about the overall high levels of in-group sharing shown in the paper to be driven by the messaging activity of a small group of extreme users that mostly follow in-group elite accounts. In Figure S4 we followed the same strategy described in the previous section to measure the ideological extremity of the ordinary users we classified as liberals and conservatives. Those with a lower score follow more out-group accounts on Twitter. We observe that the proportion of in-group sharing (proportion of shared tweets from an in-group rather than an out-group elite) is already quite high among the most moderate liberal and conservative users (around 70%), and that those users with a medium ideological extremity score (the largest group, as shown in the histogram on the top of the figure) share in-group content at a similar rate (around 90%) than those with a high score. All and all, these results indicate that high levels of in-group sharing is common among all partisan users on Twitter.

Figure S4: Proportion of ingroup sharing of tweets from elite accounts as a function of their ideological extremity.

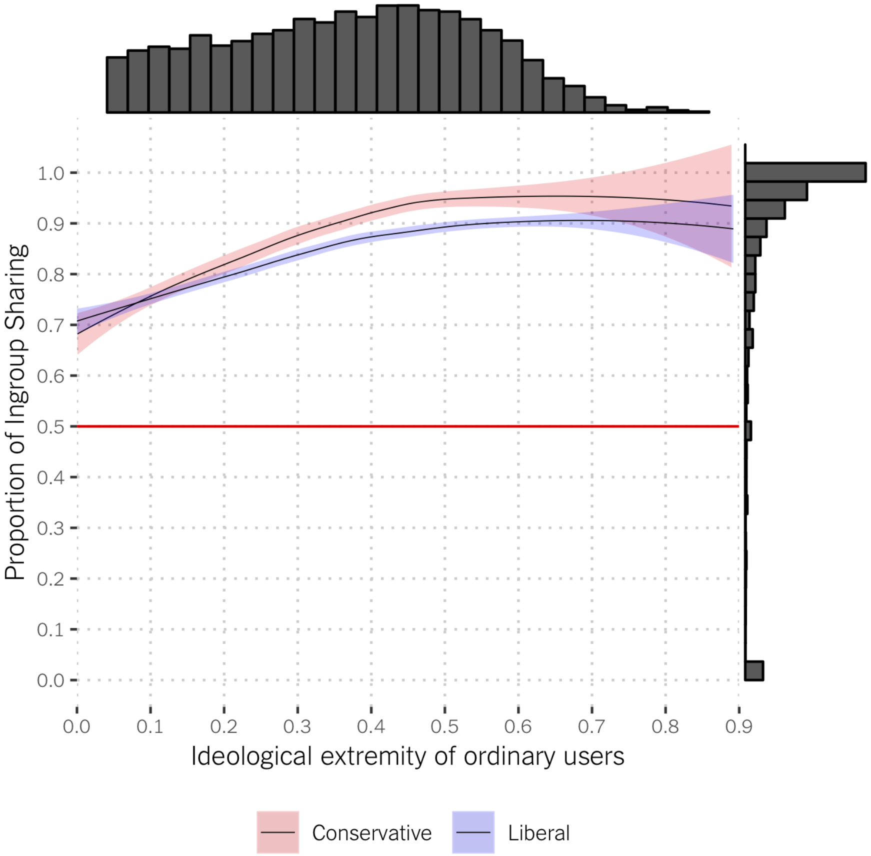

8 Sentiment classifier

First we randomly sampled about 9,000 messages from our full dataset of quote tweets, and research assistants manually coded them for whether the quote was negative, neutral or positive towards the original elite tweet. The agreement between coders was satisfactory, Krippendorff's alpha= .816. See Table S10 for examples of labeled quote tweets.

Table S10: Examples of negative, neutral, and positive comments added to original posts

Mentioned actor	Mentioned text	Added text	Attitude
RepTimRyan	We are not going back to the days of insurance companies refusing to cover those with pre-existing conditions or charging us exorbitant premiums when we get sick.	Ok try this scenero. I don't have auto insurance, then I have a wreck. I then go & buy auto insurance to pay for my auto damage. Then all the one's that have been paying for insurance all along & have no wrecks, their rates will go up to cover my wreck.	Negative
PattyMurray	Good perspective from @washingtonpost—holding the futures of thousands of young people hostage in order to advance a partisan agenda is not only cruel, but goes against everything we stand for as a nation.	Well then maybe you should stop doing that, Senator.You've had six years to get this done and haven't. Do YOUR job.	Negative
SpeakerRyan	Important that we support the nonviolent protesters in #Iran. This is the result of a regime more focused on propping up terrorist organizations than addressing the plight of its citizens.	Stop propping up the #GOP regime by having American protesters arrested and dragged out of the nation's Capitol. Capitol police have literally dragged disabled nonviolent protestors out! WTF?!? Why don't you speak up for AMERICAN protestors? #shill #Traitor #FlipItBlue	Negative
Jim_Jordan	Which floor has the exhibit about the Comey Cabal spying on the Trump campaign?	You are so funny and clever. Why don't you give up your day job and do stand up. America would be safer and we could throw tomatoes at you.	Negative
GovMikeHuckabee	I'm worried. My 1 yr old grandson is at my house. He does nothing for himself, pays for nothing, cries when he doesn't get his way, throws tantrums, and wears diapers that I have to change. I'm afraid this kid is a Democrat! Hope he outgrows it.	This is a sickening Tweet. Mr. Christian....being mean. Sick. People like @GovMikeHuckabee are causing people to run screaming from the Christian religion. THIS IS NOT a good Christian man who says stuff like this.	Negative
W7VOA	Convoy of foreign forces targeted in latest attack in capital of #Afghanistan. At least 2 fatalities reported.	U.S. forces do not appear to be among the fatalities.	Neutral
sangerkatz	Two factors have driven the opioid overdose crisis—a growing number of drug users and a deadlier drug supply. Increasingly, it's the latter that really explains the rising death toll.	Research on how benzodiazepines factor into opioid overdoses:	Neutral

nytimes	For 30 years, this photographer has been exploring the nooks and crannies of America	This photo is of my hometown. I was 8 when it was taken.	Neutral
Forbes	An American buyer purchases John Lennon's childhood home in Wavertree, Liverpool, for £480,000 (about $772,000)	@RPMRE where were you on this deal??!	Neutral
politico	With a court-ordered deadline looming for the Trump administration to reunite young immigrant children separated from their parents, the Justice Department is asking a federal judge to allow officials more time in some cases	What can be done? @mkolken	Neutral
MarkWarner	I wish the President would read these stories. We've got to make sure federal contract workers get their paychecks. We need to reopen the government.	He doesn't read.	Positive
SenToddYoung	Seniors who miss the sign-up deadline for Medicare Part B face onerous penalties that persist for the rest of their lives. Intro'd the #BENESAct to make the sign-up process more efficient and friendly for our seniors:	Thousands of people approaching #Medicare eligibility make honest mistakes in their enrollment each year. But thanks to @SenToddYoung's leadership on the #BENES Act, there is now a real opportunity to help people make decisions and avoid coverage delays and costly penalties.	Positive
RepJeffries	One by One.<br>All of the President's men are going down in flames.<br>Where there is smoke, there is often fire.<br>And there is a lot of smoke emanating from 1600 Pennsylvania Ave. right now.<br>#CleanUpCorruption	It's not a swamp, it's SPECTRE!	Positive
GOP	"Northam was talking not even about late-term abortion, he was talking about aborting an infant after it is born. That is staggering." -@tedcruz	Northam was talking about killing, resusitating, and then killing the same baby a second time! Evil! And he is a pediatrician! Is this what the Dems have become?	Positive
SenatorCollins	I talked to a waitress in Bangor this morning whose husband works for TSA. They literally had to get a loan to pay their mortgage for this month. That's just wrong. I continue to believe that a compromise is possible because this is a problem that we have to solve.	Put pressure on Senator McConnell to bring the will of the people bill that the United States House of Representatives sent to the Senate!	Positive

Then we used this annotated dataset to train the following 5 types of machine learning models predicting whether the commentaries were positive, neutral, or negative (multi-class models): (a) a Decision tree

(TREE), (b) a K-neighbors model, (c) a Support vector machine, (d) a majority-based ensemble model that took into account the output of the three previous ones, and (e) a four-layer Convolutional Neural Net. For training (a), (b), (c), and (d), we transformed all text to lowercase, removed stopwords, and lemmatized the remaining tokens to finally create a TF-IDF matrix that we used as model input. For the CNN model, we transformed all text to lower case and used 300-dimension GloVe embeddings as inputs.

We tested the accuracy of each algorithm using 5-fold crossvalidation and a 80/20 train-test split. In Figure S5 we show the overall accuracy of each of the five classifiers. The CNN proved to be the most accurate one overall. Moreover, we also assessed the ability of each classifier to predict each target category in particular. In Figure S6 we show the precision, recall, and f-score at the target class level for each of the trained classifiers. The horizontal red lines indicate the proportion of quote tweets labeled as being about each category, which serve as a key reference for judging the performance of the models: if we were to classify tweets at random, at least we would perform as well as the red line, so we should aim for the performance of our classifiers to be above the red line. We observe the CNN to score among the highest fscores for the negative and positive categories, and to do much better than the other classifiers in predicting the neutral category. For this reason we decided to used the CNN classifier to predict the tone of all quote tweets in our dataset.

Figure S5: Overall out-of-sample accuracy of the 5 machine learning classifiers trained to predict the tone of the quote tweets.

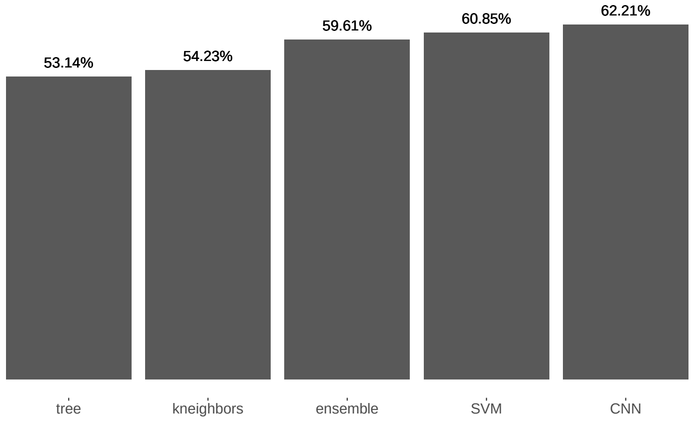

We note that our labeling did not look at whether the political elites analyzed engaged in costly talk (e.g., Liz Cheney tweeting that Donald Trump lost the election and a liberal users quote retweeting it). Such an example presents various complexities (e.g., it a quote tweet negative toward the out-group if it praises the purpose of the original tweet? Would a quote tweet that said something like, "Even a monster like Liz Cheney can see that Trump lost" be coded: positive, negative or neutral?). The coding scheme did not account for cases in which a partisan elite criticizes another in-group elite and a user retweets this with complex reactions. The research assistants would probably code this instance as either neutral (i.e., positive and negative cancel each other out) or positive (in that what the users tried to say is that even a bad person is doing something right this time). In general, however, we believe that such cases are very rare in our dataset and none of the research assistants brought such questions to the training and validation meetings. We acknowledge that we cannot estimate how many costly talk posts there are and how they are coded in our data. Given the way that some costly talk posts are quickly policed by other followers in the in-group, this is a limitation, which – however – is unlikely to substantively change any patterns presented.

Figure S6: Target-class-level precision, recall and f-score for the 5 machine learning classifiers trained to predict the tone of the quote tweets.

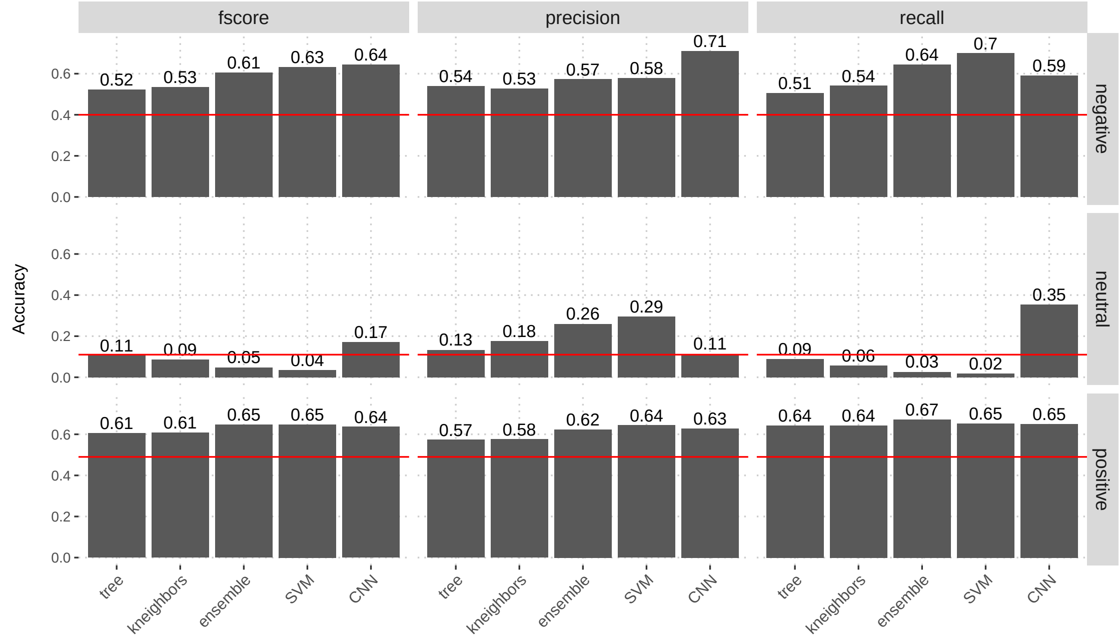

9 Topic classifier

In this section we explain how we automatically coded the topic of the content of the original tweets from elite accounts that we study. Given the large number of tweets, manual coding was not practical for the full corpus. Instead, we trained a machine learning model (a Convolutional Neural Net (CNN)) predicting whether each tweet discussed one of the 20 topics of the Comparative Agendas Project (CAP) (*66*), a comprehensive and widely used classification for studying political agendas, plus a non-policy-issue category reserved for tweets that did not address any substantive policy area, such as tweets commemorating holidays. See Table S13 for a list of topics. We excluded *Culture* topics from the 21 topics of the CAP codebook because in initial tests we saw very low numbers of tweets about this topic and the topic was not very relevant for the analysis carried out here.

Similar to (*51*), we trained a three-layer CNN. Figure S7 illustrates the architecture used to identify CAP-topics. We next describe our two main model architectures, and then proceed to describe in detail our training data-sets and valdation scheme. This model was originally trained and validated for another of our projects in which we needed to predict the topic of tweets sent by state legislators in the United States around the same time period, so we are confident that it does a good job at predicting the topic of tweets sent by political elites.

First, we represented each word in a given sentence as a 300-dimension word- embedding (a vector that ideally represents an integration of each word's meaning and context/position in the text as dense features for further analysis) (*67*). We obtained the models used to produce word-embeddings by finetuning a pretrained Word2Vec model for an additional 10 epochs (*68*), to which we had first added all unique new vocabulary present in our training datsets as well as in the tweets to which we wanted to apply the resulting model. We used the python `Gensim` word2vec model and methods, and GloVe pretrained embeddings: Common Crawl (42B tokens, 1.9M vocab, uncased, 300d vectors, 1.75 GB download).

This results in a three-dimensional matrix ($n \times k \times d$) that is used as our primary model input, where n is the maximum word length for all training documents, k is the size of the embedding (300), and d is the

number of documents to pass through to the CNN.

Figure S7: Architecture of the Convolutional Neural Net predicting the policy topics discussed in tweets by state legislators.

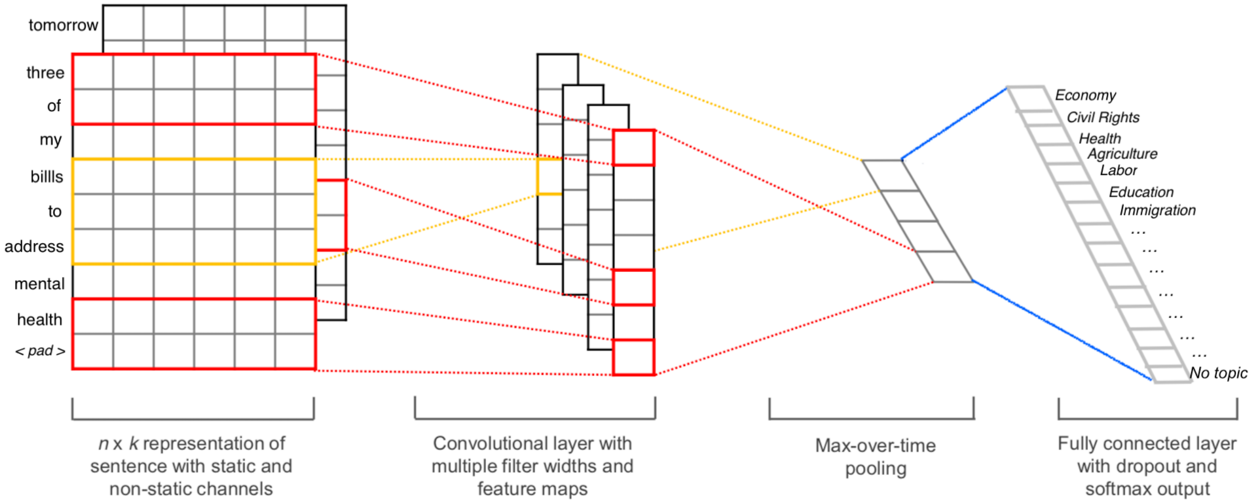

The CNN that comprises our second model (the model used to classify the CAP topic) has three convolutional layers of different sizes, each processing 3-, 4-, and 5-word embeddings at a time, and so producing hidden layers of different sizes. These hidden layers are joined into a single vector for each document by max-pooling the weights in each word-vector. The last stage of the CNN is comprised of a fully connected layer mapping the previous max-pooled vector to the 21 CAP issue classes (20 policy areas plus the "non-policy/not-relevant" class). We employ a cross-entropy loss function; gradient optimization is performed via adaptive moment estimation (*69*). We use a batch size of 64 for training the model.

We trained the model with various datasets, assessed the out-of-sample accuracy of each model-dataset pair and selected the best performing model-data pairing to generate topic predictions for tweets sent by political elites in 2018: state legislators from the United States. In our training datasets, each observation (document or tweet) has been coded as belonging to one (mutually exclusive) topic category (or the no-topic one).

We used four datasets, described in Table S11. The first one is composed of publicly available data, the second one comes from the replication material of a published study, and the final two have been created/annotated by us for the purpose of this and other studies. In the first dataset (A) we combined all available CAP-labeled datasets for the United States available in the CAP website (789,004 observations in total). The second dataset (B) is comprised of 45,394 tweets from Senators who served during the 113th Congress and that were labeled by (*70*). The third set (C) consists of 18,088 tweets sent by media accounts and followers of state legislators that we coded according to the CAP classification. The fourth dataset (D) consists of 3,368 tweets sent by the state legislators that we also annotated. A total of six coders (research assistants) participated in the annotation of sets C and D. Two coders annotated the tweets sent by media accounts (C.a): 89% agreement and 0.7 Cohen's Kappa. Two other coders annotated the tweets sent by followers of state legislators (C.b): 91% agreement and 0.77 Cohen's Kappa. And finally, a different pair of coders annotated the tweets sent by state legislators (D): 87.1% agreement and 0.74 Cohen's Kappa. We trained the same CNN model nine times using the following data combinations, with the goal of taking advantage of transfer learning and training more accurate models than simply training the model with the tweets from state legislators that we had coded (so only set D): (1) only set A, (2) only set D, (3) set A and set D, (4) set D and a small sample of set A (1,300 observations), (5) set D and a smaller sample of set A (650 observations), (6) set D and set B, (7) set D and a small sample of set B (1,300 tweets), (8) set D and a smaller sample of set B (650 tweets), (9) set D and set C.

Table S11: Public datasets coded using the CAP 21-issue classification, used for training and testing a classifier predicting *Policy Issues* in tweets from state legislators.

Set	Dataset	Time	N
A	Congressional Quarterly Almanac	1948-2015	14,444
	New York Times Front Page	1996-2006	31,034
	New York Times Index	1946-2014	54,578
	Congressional Bills	1947-2016	463,762
	Congressional Hearings	1946-2015	97,593
	Public Law Titles	1948-2011	33,644
	Public Laws	1948-2017	20,928
	Executive Orders	1945-2017	4,294
	Presidential Veto Rhetoric	1985-2016	1,618
	State of the Union Speeches	1946-2018	22,289
	Democratic Party Platform	1948-2016	15,953
	Republican Party Platform	1948-2016	19,836
	Supreme Court Cases	1944-2009	9,031
B	Tweets sent by Senators 113th Congress	2013-2015	45,394
C	Tweets sent by media accounts	2018	8,802
	Tweets sent by followers of state legislators	2018	9,286
D	Tweets sent by state legislators	2018	3,368
	Total	1944-2018	855,854

Table S12: Out of sample accuracy of the nine versions of the CNN model we trained predicting the political topics of the Comparative Agendas Project.

Model version	Test Accuracy	Test Accuracy policy tweets
(6) set D and B	0.78	0.79
(1) set A	0.73	0.73
(3) set D and A	0.73	0.73
(9) set D and C	0.77	0.49
(7) set D and small B	0.56	0.36
(4) set D and small A	0.55	0.32
(8) set D and smaller B	0.57	0.29
(5) set D and smaller A	0.56	0.28
(2) set D	0.60	0.26

To assess the performance of these nine versions of the model we split the data used in each case into a train and test set. In Table S12 we report the accuracy of the nine versions of the model we trained (based on 3-fold cross-validation), based on held-out test sets. We assess the test accuracy when predicting all tweets in the test split (*Test Accuracy*), and also when only predicting the tweets coded as being about one of the policy areas, so after excluding the no-policy tweets (*Test accuracy policy tweets*). The tweets not related to any policy area represented a large part of the tweets we coded from state legislators (set D) and we wanted to make sure that our model did well at both distinguishing overall policy relevance and at distinguishing between policy areas.

The model trained with the coded tweets by state legislators plus the coded tweets sent by Senators of

Table S13: Class accuracy and f-score for the best performing model

Policy Area	Class Proportion	Test Accuracy	Test F-score
No Policy	0.48	0.74	0.82
Govt. Operations	0.13	0.81	0.75
Health	0.06	0.80	0.73
Economy	0.05	0.74	0.76
Education	0.03	0.83	0.67
Civil Rights	0.03	0.69	0.58
Housing	0.02	0.36	0.46
Environment	0.02	0.58	0.61
Transportation	0.02	0.73	0.68
Agriculture	0.02	0.93	0.72
Energy	0.02	0.83	0.70
Social Welfare	0.02	0.53	0.61
Law & Crime	0.02	0.67	0.48
Intl. Affairs	0.01	0.71	0.63
Immigration	0.01	0.92	0.92
Public Lands	0.01	0.67	0.63
Labor	0.01	0.70	0.56
Domestic Commerce	0.01	0.75	0.45
Technology	0.01	0.38	0.50
Defense	0.00	0.75	0.35

the 113th Congress returned the best (and very satisfactory) results when predicting out of sample the topic of tweets from state legislators. We hence used this model to generate topic predictions for the tweets sent by the elite accounts in the paper. The test accuracy in both cases is close to 80% (very high given that the model is predicting 21 topic classes, and that topic overlap is possible in the training set). In table S13 we show that the accuracy and f-score (based on the held-out test sets) is very high for all the topic classes, despite most of them being rarely discussed.

To assess the face validity of the model, in Table S14 we show the top distinctive text features of the tweets predicted to be about each topic. Reassuringly, the top features seem to be relevant to each topic.

Table S14: Top distinctive features of tweets predicted by the CNN to be about each topic or policy area.

Topic	Top Features
No policy issue	thank, great, all, today, so, day, me, one, happy, m, good, time, thanks, join
Healthcare	health, care, maternal, mental, new, day, flu, healthcare, work, get, state, medicaid, mortality, all
Gov. Operations	vote, election, today, day, state, voting, primary, early, all, house, time, democratic, people
Intl. Affairs	russia, russian, putin, right, now, trumps, world, american, dead, peace, people, says
Public Lands	park, state, fire, during, public, thank, wildlife, beautiful, city, confederate, contained, discussion, grand, heritage, history, land
Labor	job, workers, fair, jobs, community, employees, need, work, workforce, working, youth, according, better
Law and Crime	children, gun, violence, families, law, parents, sexual, separated, thank, guns, people, child, school, community
Defense	military, veterans, war, one, day, women, state, families, honor, veteran, friend
Immigration	daca, children, border, immigration, immigrants, immigrant, families, dreamers, policy, parents, detention, migrant, family, stand, trumps
Domestic Commerce	harvey, flooding, community, business, small, city, need, local, businesses, hurricane, many, disaster, state, flood
Civil Rights	women, rights, scotus, redistricting, day, voter, case, join, people, court, families
Economy	tax, taxes, property, budget, economy, state, tariffs, need, spending
Environment	water, climate, change, earthday, right, air, cleanup, lake, nasa, san, brownsville, great, mars
Transportation	transportation, nasa, outhwestair, airlines, future, hearing, bus, get
Energy	energy, gas, oil, solar, texasoilnews, oilandgas, texasoil, txenergy, back, coal, committee, cpsenergy, nasa, natural, next
Agriculture	food, farm, farmers, agriculture, taking, agricultural, bureau, campus, farming, learning
Social Welfare	food, safoodbank, snap, hunger, meals, help, million,free, program, children, kids, nutritious, summer
Education	school, students, education, public, schools, teachers, state, finance, college, high, teacher, funding, kids
Technology	nasa, space, station, mission, astronaut, international, students, media, crew, launch, satellite, astronauts, awards, contract, earth, internet
Foreign Trade	trade, trumps, war, abandons, barrel, beijing, billion, breath, canada, chinese, cover, currently, deals, economy
Housing	housing, affordable, hearing, association, austin, citys, gentrification, losaltos, meeting, neighborhood, policy, access, aff, affairs

10 Descriptives on how many elite accounts ordinary users follow

In the following figure we show the distribution of how many elite accounts our sample of 1,437,774 ordinary users follow; out of the list of 2,624 elite accounts (489 politicians, 2,016 pundits, and 119 media organizations). We show that most of the users (60%) do not follow any of these politically relevant elites, that only around 30% follow more than 2, and a small minority (2.5%) follows many (> 50 elite accounts).

In generating the random sample of 1,437,774 users to track, we did not set up any geolocation filter. We increased the likelihood of our users being from the United States by using a set of English stopwords when

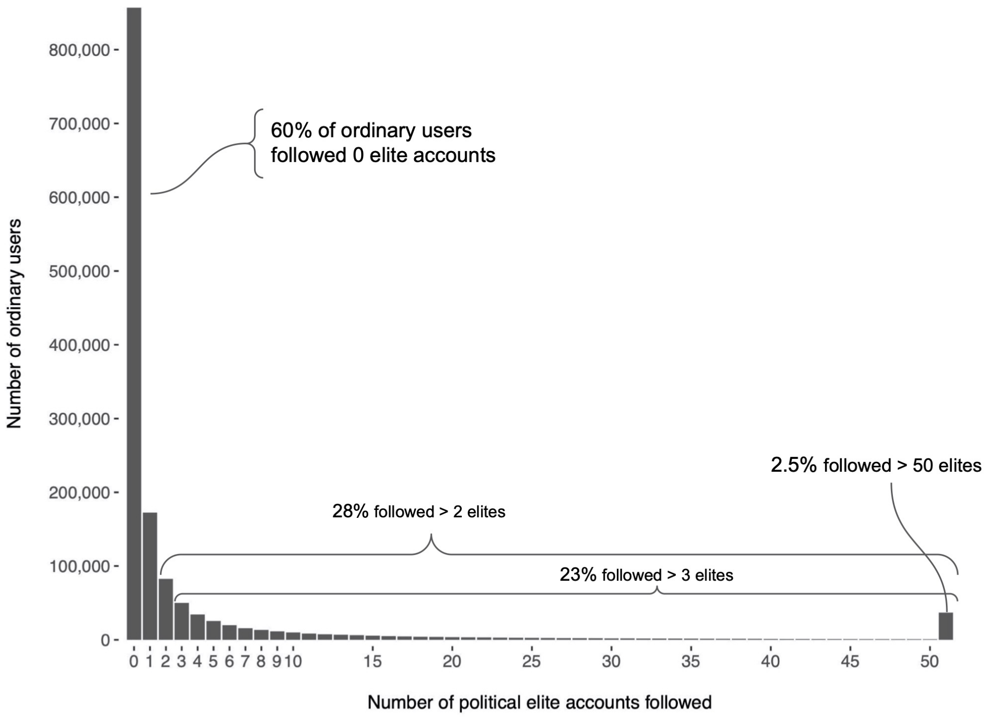

Figure S8: Number of political elite accounts followed by 1,437,774 ordinary Twitter users.

collecting the random messages and users, and by collecting at a time where US users are likely to be active *vs.* users from other English-speaking countries. However, to ensure that the findings on the proportion of users that follow none *vs.* particular number of elite accounts are indeed generalizable to Twitter users from the US, we leverage a list of users from our random sample (N = 24,328) that we had located in the US for another study (see SI.B in (*12*)). In Table S15 we report the proportion of users of this sample that we have confidently located in the US that follow 0, 1, 2, etc. elite accounts in our sample. Consistent with the main findings reported in the paper, we find that around 60% of them do not follow any of the elite accounts (journalists, media, and politician) in our list.

Table S15: Number of elite accounts followed by 24,328 we have confidently located in the United States.

Elite accounts followed	Number of users	Percentage of users
0	14643	60.20
1	3405	14.00
2	1545	6.40
3	915	3.80
4	591	2.40
5	455	1.90
6	366	1.50
7	261	1.10
8	193	0.80
9	179	0.70
10	156	0.60
10+	1619	6.70

11 Exploring overlap in following, sharing and annotation patterns, between Liberals and Conservatives

In the paper we show how the ordinary users from our large sample (1,437,775) that we have classified as liberals (115,589) and conservatives (35,474), they follow, share, and positively comment at a much higher rates in-group elite accounts, compared to out-group elite accounts. However, recent studies find a substantial overlap in the kinds of media outlets and politicians people on average consume/follow (*16, 48*).

We believe that this argument and the findings in the paper are not necessarily contradictory. In our study we look at how ordinary users engage with politically-relevant elites on Twitter, with a particular focus on whether ordinary users are more likely to engage with in- *v.* out-group elites on a range of behavioral measures. Although we do pay attention to our full sample (1,437,775) when we assess how many engage at all with the accounts in our list of 2,624 elite accounts; for the rest of the analysis (focused on in-/out-group dynamics) we need to constrain ourselves to assessing those users that follow enough elite accounts from Barbera and colleagues' work (*12*) and so for which we can get an ideology score (180,203). In addition, to make sure we can clearly identify the out-group of each user, we have further constrain our analysis to those users we classified as liberals and conservatives; excluding 29,140 moderates.

In turn, this means that our analysis in the paper focuses on those users that have a more clearly defined ideological group – for which existing research (*16, 48*) also finds that their media and political diets are less likely to overlap with those holding opposing views.

To speak more clearly to such literature, in Figure S9 we run the following analyses for those we classified as liberals and conservatives (breaking each down into two groups, for further analytical nuance), as well as those we classified as moderates. First, in the most left panel we show the percentage that elite accounts (x-axis) from each ideological group represents of all the elite accounts followed by each ideological group of ordinary users (facet titles). Three clear takeaways emerge from this sub figure. First, if we look at the percentage of moderate elite accounts each ideological group of ordinary users follows, we see that there is some overlap in terms of elite following (e.g. 36% of the elites Liberal users follow and 21.2% for Conservative). Second, overall we still see a clear in-group balance for those ordinary users we classified as liberals ('Liberals' and 'Liberals +' in Figure S9). For example, even when taking into account whether they follow Moderate elites, 55% of the elite accounts followed by ordinary Liberal users are liberal accounts (3.6% + 51.9%), and only 8.5% are conservative elite accounts (6.4% + 2.1%). Finally, in line with what we observe in the paper, despite being quite low, following out-group accounts is slightly higher for conservative users.

In the middle panel of Figure S9 we replicate the same analysis but this time we focus on the sharing behavior (retweets). There are a couple findings that we would like to emphasize. First, also in line with what we observe in the paper, even when including moderate users and elites into the analysis, we see the amount of in-group (v. out-group) engagement to increase, compared to the following behavior. For example, Liberal elite accounts represent 51.9% of the accounts followed by Liberal users, but they represent 57.6% of the tweets from elite accounts they retweet. Hence, in turn, the overlap between the behavior of liberal and conservative users decreases, compared to their following behavior. Moderate elite accounts represent 36% and 21.2% of all the elite accounts Liberal and Conservative users follow (respectively), yet they only represent 33.4% and 7.1% of the tweets from elite accounts they retweet.

Finally, in the most right panel, we explore the annotation behavior of these same liberal, conservative and moderate users. The sub figure shows how more/less likely the ordinary users in that ideological group (facet title) are to add a negative (v. neutral or positive) comment when quote tweeting a message from an elite account from that ideological group (x-axis). Although all these groups of ordinary users and fairly equally likely to criticise v. praise tweets from Moderate elites (maybe with the exception of 'Conservative +' users), we do see how much more likely they are to negatively annotate tweets sent by out-group elites. This is in line with what we show in the paper (although in there we show predicted values from a multivariate model, Fig. 4, while in here we focus on simple bivariate descriptives), and it emphasizes that although there is some behavioral overlap between liberals and conservatives (especially when it comes to elite following), even when we include moderates in the overlap exploration, we still find clear in-group biases, especially

when we look at more 'active' type of behaviors, such as sharing and annotating. As a final note, as we emphasize in the paper, the ordinary users we classified into liberals and conservatives (so 'Liberal +', 'Liberal', 'Conservative', and 'Conservative +' in Figure S9) only represent 13% of the accounts in our full sample, but they represent 86% of all the retweets and quote tweets for the elite accounts in our list. So in-group biases for these groups of users can have a large effect on the kind of content ordinary users are exposed to and engage with.

Figure S9: **Left and Center panel:** Proportion that elite accounts from particular ideological groups (x-axis) represent out of all elite accounts followed (left panel) and shared (center panel) by ordinary users from different ideological groups (facet titles). **Right panel:** How more/less likely ordinary users from different ideological groups (facet titles) are to add negative (v. neutral or positive) quotes to tweets originally sent by elite accounts from different ideological groups (x-axis).

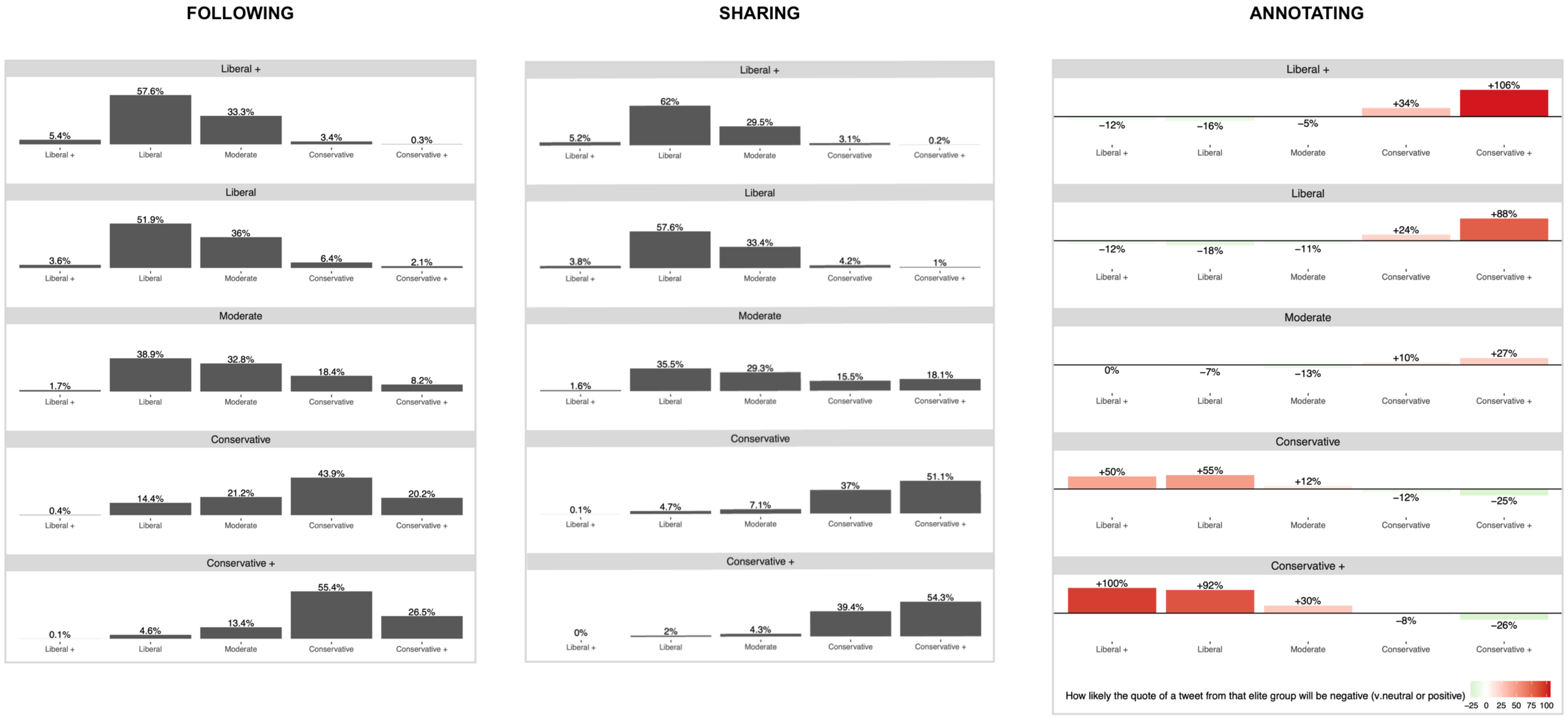

12 If they don't follow many politically-relevant accounts, who do they follow? Comparing the amount of politically-relevant accounts v. celebrity accounts that Twitter users follow

One of the key findings of the paper is that a substantial proportion of the full sample of 1,437,775 Twitter users we study doesn't follow any, or very few, of the 2,624 politically-relevant accounts (journalists, media, and politician accounts) we study (e.g. 60% follow none of them). If they don't follow many politically-relevant accounts, who do they follow? We wanted to compare the amount of political elite accounts followed to some baseline, so we checked how often they follow accounts of celebrities (musicians, athletes, actors, tv stars, business man/women etc.) v. the journalists, media and politician accounts in our analysis. To conduct this analysis, we combine two open-source datasets of celebrities: this one with the 1,000 most followed elites on Twitter (`https://gist.github.com/mbejda/9c3353780270e7298763`), and another one of 86 celebrities (*71*) (`https://github.com/zilinskyjan/cultural-elites/blob/master/data/cultural_endorsements_2016.csv`). After merging them, removing duplicates, and checking which Twitter accounts are still active, we ended up with a final sample of 971 celebrities. To run this analysis we had to collect the full list of accounts each user in our sample follows (which requires a very long time to collect using the Twitter API). Hence we selected a random sample of about half of the size (N = 720,555) for which we conducted the analysis. Please note that for the analysis we conduct in the paper, we relied on information we had collected about the followers of each of the politically relevant accounts we study. However this approach was off the table for exploring the most followed celebrities on Twitter, as it would have taken an incredibly long time to collect their full list of followers using the Twitter API.

Figure S10: Percentage of users who follow at least a particular number of celebrity, journalist, media, and politician accounts.

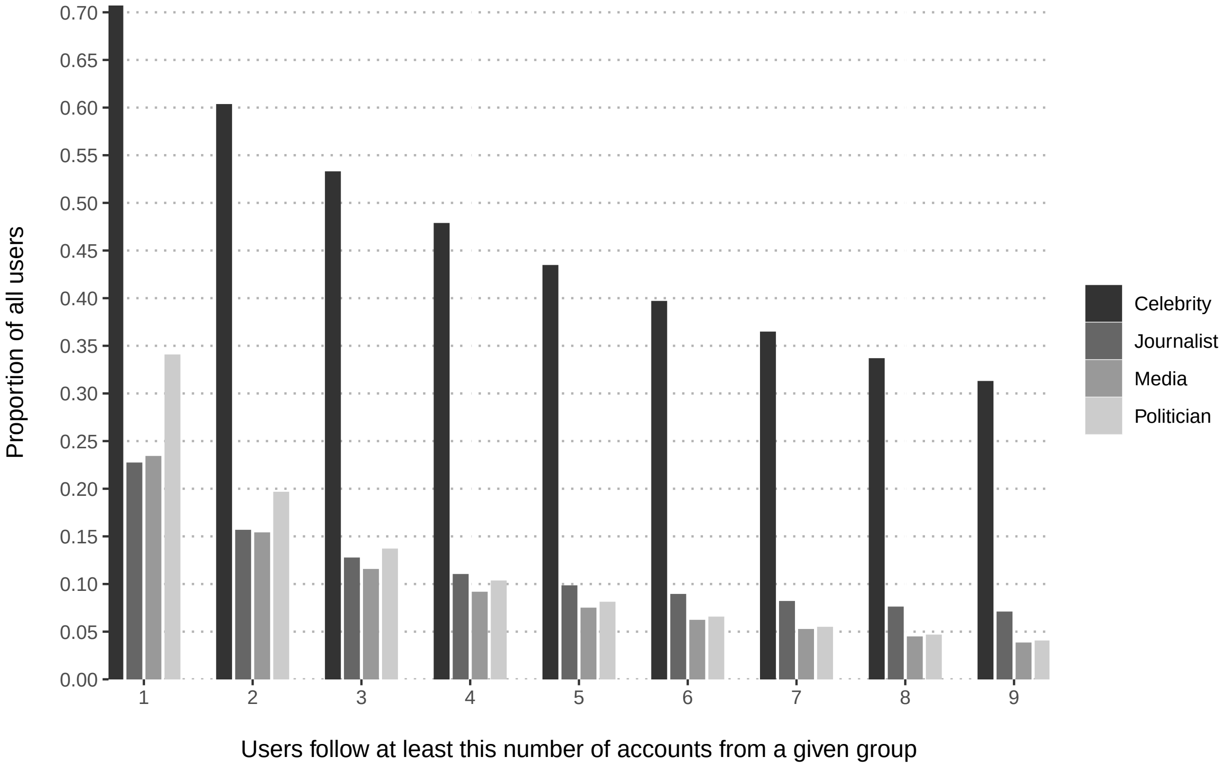

In Figure S10 we show the percentage of users in our study who follow at least a given number of celebrity, journalist, media, and politician accounts. The difference between celebrities and the other three groups of accounts is substantial. For example, at least about 70% of them follow at least 1 celebrity, yet only about 35% follow a politician, 24% a media account, and 23% a journalist or media pundit. More than 50% of the users follow at least 3 celebrities, while less than 14% follows 3 journalists, or 3 media, or 3 politician accounts.

In Table S16 we provide information about, on average, how many celebrities, journalists, media and politicians the users in our sample follow (with 95% confidence intervals). Again we see that they follow celebrities at a much larger rate (10.7 on average), followed by journalists (3.35), politician (1.52), and media accounts (1.13). The top-5 most followed celebrities by the users in our sample are Ellen DeGeneres (followed by 12.3% of the users), Rihanna (10.2%), Elon Musk (9.9%), Jimmy Fallon (9.4%), and Bill Gates (9%).

Table S16: Average number of celebrity, journalist, politician, and media accounts followed by the Twitter users in our sample.

Group	Avg. number of accounts followed [95% conf. int.]
Celebrity	10.7 [10.65-10.74]
Journalist	3.35 [3.31-3.39]
Politician	1.52 [1.51-1.53]
Media	1.13 [1.13-1.14]

In sum, given by how many follow celebrities, this analysis shows that the ordinary Twitter users in our sample do use the platform to engage with others beyond their family and friends; but only a small proportion decide to engage with politically-relevant accounts.

13 Patterns of in-/out-group sharing when including the moderate users and elites in the analysis.

In Figure 2 of the paper, we explore how often users share (and annotate) tweets from in- compared to out-group political elites. To run such analysis, we first use Barbera and colleagues' (*12*) method to identify liberal and conservative users (151,063 out of the 180,203 for which we had an ideology score) and elites (1,721 out of the 2,624 elites in the full list of journalists, media, and politician accounts). We exclude from the analysis 29,140 users and 903 elite accounts classified as moderates, as they lack a clear out-group. In this Appendix we show that the results reported in Figure 2 of the paper are robust to including these moderate users into the analysis (by classifying them into liberals and conservatives). We classified as moderates those with an ideology score from Barbera and colleagues' (*12*) method between 0 and 1.2. For this additional analysis we classified as liberals all users and actors with a score below 0.7, and as conservatives those with a score above 0.7.

We observe the main findings from Figure 2 to hold when including the moderates into the analysis. Ingroup sharing is still very high (86.3% vs. 13.5% out-group), users are more likely to add a commentary when sharing a message from an out-group account (22.6% vs. 8.4% for in-group), and such commentary is more likely to be negative when sharing an out-group message.

Figure S11: How often users share (annotate, and negatively annotate) tweets from in-/out-group elie accounts. Comparing the results when we include or exclude the moderates users and elite accounts in our sample.

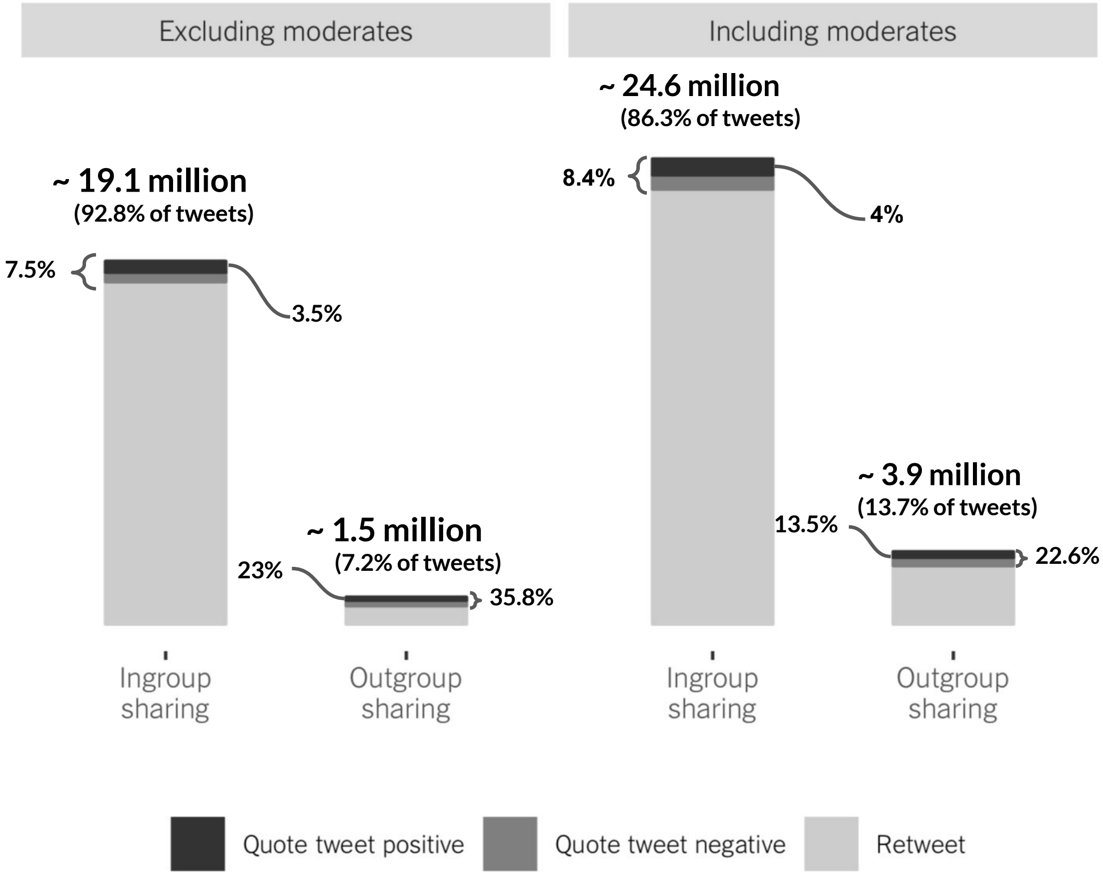

REFERENCES AND NOTES

1. R. Fletcher, R. K. Nielsen, Are people incidentally exposed to news on social media? A comparative analysis. *New Media Soc.* **20**, 2450–2468 (2018).

2. A. W. Geiger, *Key Findings About the Online News Landscape in America* (2019).

3. E. Pariser, *The Filter Bubble: How the New Personalized Web Is Changing What We Read and How We Think* (Penguin Books, reprint edition, 2012).

4. C. R. Sunstein, *Republic: Divided Democracy in the Age of Social Media* (Princeton Univ. Press, 2018).

5. E. J. Finkel, C. A. Bail, M. Cikara, P. H. Ditto, S. Iyengar, S. Klar, L. Mason, M. C. McGrath, B. Nyhan, D. G. Rand, L. J. Skitka, J. A. Tucker, J. J. Van Bavel, C. S. Wang, J. N. Druckman, Political sectarianism in America. *Science* **370**, 533–536 (2020).

6. M. J. Hetherington, T. J. Rudolph, *Why Washington Won't Work: Polarization, Political Trust, and the Governing Crisis* (University of Chicago Press, illustrated edition, 2015).

7. S. Iyengar, Y. Lelkes, M. Levendusky, N. Malhotra, S. J. Westwood, The origins and consequences of affective polarization in the united states. *Annu. Rev. Polit. Sci.* **22**, 129–146 (2019).

8. N. Persily, J. A. Tucker, Eds., *Social Media and Democracy: The State of the Field, Prospects for Reform* (Cambridge Univ. Press, 2020).

9. P. Barberá, Social media, echo chambers, and political polarization, in *Social Media and Democracy: The State of the Field, Prospects for Reform*, N. Persily, J. A. Tucker, Eds. (Cambridge Univ. Press, 2020).

10. L. A. Adamic, N. Glance, The political blogosphere and the 2004 US election: Divided they blog, in *Proceedings of the 3rd International Workshop on Link Discovery* (ACM, 2005), pp. 36–43.

11. E. Lawrence, J. Sides, H. Farrell, Self-segregation or deliberation? Blog readership, participation, and polarization in American politics. *Perspect. Polit.* **8**, 141–157 (2010).

12. P. Barbera, J. T. Jost, J. Nagler, J. A. Tucker, R. Bonneau, Tweeting from left to right: Is online political communication more than an echo chamber? *Psychol. Sci.* **26**, 1531–1542 (2015).

13. G. Rivero, Preaching to the choir: Ideology and following behaviour in social media. *Contemp. Soc. Sci.* **14**, 54–70 (2019).

14. E. Colleoni, A. Rozza, A. Arvidsson, Echo chamber or public sphere? Predicting political orientation and measuring political homophily in twitter using big data. *J. Commun.* **64**, 317–332 (2014).

15. A. Boutet, H. Kim, E. Yoneki, What's in your tweets? I know who you supported in the UK 2010 general election. *Proc. Intl. AAAI Conf. Web Soc. Media* **6**, 411–414 (2012).

16. G. Eady, J. Nagler, A. Guess, J. Zilinsky, J. A. Tucker, How many people live in political bubbles on social media? Evidence from linked survey and twitter data. *SAGE Open* **9**, 2158244019832705 (2019).

17. W. Chen, D. Pacheco, K.-C. Yang, F. Menczer, Neutral bots probe political bias on social media. *Nat. Commun.* **12**, 5580 (2021).

18. M. D. Conover, B. Gonzalves, A. Flammini, F. Menczer, Partisan asymmetries in online political activity. *EPJ Data Sci.* **1**, 6 (2012).

19. P. Barbera, A. Casas, J. Nagler, P. J. Egan, R. Bonneau, J. T. Jost, J. A. Tucker, Who leads? Who follows? Measuring issue attention and agenda setting by legislators and the mass public using social media data. *Am. Polit. Sci. Rev.* **113**, 883–901 (2019).

20. G. Stocking, M. Barthel, E. Grieco, *Sources Shared on Twitter: A Case Study on Immigration* (2018).

21. P. W. Kraft, Y. Krupnikov, K. Milita, J. B. Ryan, S. Soroka, Social media and the changing information environment: Sentiment differences in read versus recirculated news content. *Public Opin. Q.* **84**, 195–215 (2020).

22. J. N. Druckman, E. Peterson, R. Slothuus, How elite partisan polarization affects public opinion formation. *Am. Polit. Sci. Rev.* **107**, 57–79 (2013).

23. K. J. Mullinix, Partisanship and preference formation: Competing motivations, elite polarization, and issue importance. *Polit. Behav.* **38**, 383–411 (2016).

24. S. P. Nicholson, Polarizing cues. *Am. J. Polit. Sci.* **56**, 52–66 (2012).

25. M. Levendusky, *How Partisan Media Polarize America* (University of Chicago Press, 2013).

26. M. Levendusky, N. Malhotra, Does media coverage of partisan polarization affect political attitudes? *Polit. Commun.* **33**, 283–301 (2016).

27. P. Metaxas, E. Mustafaraj, K. Wong, L. Zeng, M. O'Keefe, S. Finn, What do retweets indicate? Results from user survey and meta-review of research. *Proc. Intl. AAAI Conf. Web Soc. Media* **9**, 658–661 (2015).

28. L. Huddy, Contrasting theoretical approaches to intergroup relations. *Polit. Psychol.* **25**, 947–967 (2004).

29. H. Tajfel, Social psychology of intergroup relations. *Annu. Rev. Psychol.* **33**, 1–39 (1982).

30. H. Tajfel, J. Turner, An intergrative theory of intergroup conflict, in *Organization Identity: A Reader*, M. J. Hatch, M. Schultz, Eds. (Oxford Univ. Press, 1979), pp. 56–66.

31. M. B. Brewer, Ingroup identification and intergroup conflict, in *Social Identity, Intergroup Conflict, and Conflict Reduction*, R. D. Ashmore, L. Jussim, D. Wilder, Eds. (Oxford Univ. Press, 2001), pp. 17–41.

32. M. A. Baum, T. Groeling, Shot by the messenger: Partisan cues and public opinion regarding national security and war. *Polit. Behav.* **31**, 157–186 (2009).

33. R. Puglisi, J. M. Snyder, Newspaper coverage of political scandals. *J. Polit.* **73**, 931–950 (2011).

34. J. M. Berry, S. Sobieraj, *The Outrage Industry: Political Opinion Media and the New Incivility* (Oxford Univ. Press, 2013).

35. A. Feller, M. Kuhnert, T. Sprenger, I. Welpe, Divided they tweet: The network structure of political microbloggers and discussion topics. *Proc. Intl. AAAI Conf. Web Soc. Media* **5**, 474–477 (2011).

36. E. Colleoni, A. Rozza, A. Arvidsson, Echo chamber or public sphere? Predicting political orientation and measuring political homophily in twitter using big data: Political homophily on twitter. *J. Commun.* **64**, 317–332 (2014).

37. C. Mcclain, R. Widjaya, G. Rivero, A. Smith, *The Behaviors and Attitudes of U.S. Adults on Twitter* (2021).

38. L. Mason, *Uncivil Agreement: How Politics Became Our Identity* (University of Chicago Press, 2018).

39. C. S. Taber, M. Lodge, Motivated skepticism in the evaluation of political beliefs. *Am. J. Polit. Sci.* **50**, 755–769 (2006).

40. M. Osmundsen, A. Bor, P. B. Vahlstrup, A. Bechmann, M. B. Petersen, Partisan polarization is the primary psychological motivation behind political fake news sharing on twitter. *Am. Polit. Sci. Rev.* **115**, 999–1015 (2021).

41. S. Rathje, J. J. Van Bavel, S. van der Linden, Out-group animosity drives engagement on social media. *Proc. Natl. Acad. Sci. U.S.A.* **118**, e2024292118 (2021).

42. E. Bakshy, S. Messing, L. A. Adamic, Exposure to ideologically diverse news and opinion on Facebook. *Science* **348**, 1130–1132 (2015).

43. N. Bartley, A. Abeliuk, E. Ferrara, K. Lerman, Auditing algorithmic bias on twitter, in *Proceedings of the 13th ACM Web Science Conference 2021* (ACM, 2021), pp. 65–73.

44. M. Zuckerberg, *Facebook* (2018); www.facebook.com/zuck/posts/10104445245963251.

45. C. Wells, K. Thorson, Combining big data and survey techniques to model effects of political content flows in facebook. *Soc. Sci. Comp. Rev.* **35**, 33–52 (2017).

46. S. Flaxman, S. Goel, J. M. Rao, Filter bubbles, echo chambers, and online news consumption. *Public Opin. Q.* **80**, 298–320 (2016).

47. M. Wojcieszak, S. de Leeuw, E. Menchen-Trevino, S. Lee, K. M. Huang-Isherwood, B. Weeks, No polarization from partisan news: Over-time evidence from trace data. *Intl. J. Press/Politics* 10.1177/19401612211047194 (2021).

48. A. Guess, (Almost) everything in moderation: New evidence on Americans' online media diets. *Am. J. Polit. Sci.* **65**, 1007–1022 (2021).

49. J. Allen, B. Howland, M. Mobius, D. Rothschild, D. J. Watts, Evaluating the fake news problem at the scale of the information ecosystem. *Sci. Adv.* **6**, eaay3539 (2020).

50. N. Grinberg, K. Joseph, L. Friedland, B. Swire-Thompson, D. Lazer, Fake news on twitter during the 2016 U.S. presidential election. *Science* **363**, 374–378 (2019).

51. Y. Kim, Convolutional neural networks for sentence classification, in *Proceedings of the 2014 Conference on Empirical Methods in Natural Language Processing (EMNLP)* (Association for Computational Linguistics, 2014), pp. 1746–1751.

52. D. J. Hopkins, *The Increasingly United States: How and Why American Political Behavior Nationalized* (University of Chicago Press, 2018).

53. P. M. Fernbach, L. Van Boven, False polarization: Cognitive mechanisms and potential solutions. *Curr. Opin. Psychol.* **43**, 1–6 (2022).

54. J. A. Tucker, A. Guess, P. Barbera, C. Vaccari, A. Siegel, S. Sanovich, D. Stukal, B. Nyhan, Social Media, Political Polarization, and Political Disinformation: A Review of the Scientific Literature, SSRN Scholarly Paper ID 3144139, Social Science Research Network, Rochester, NY (March 2018).

55. W. J. Brady, J. A. Wills, D. Burkart, J. T. Jost, J. J. Van Bavel, An ideological asymmetry in the diffusion of moralized content on social media among political leaders. *J. Exp. Psychol. Gen.* **148**, 1802–1813 (2019).

56. J. T. Jost, J. Glaser, A. W. Kruglanski, F. J. Sulloway, Political conservatism as motivated social cognition. *Psychol. Bull.* **129**, 339–375 (2003).

57. C. A. Bail, L. P. Argyle, T. W. Brown, J. P. Bumpus, H. Chen, M. B. Fallin Hunzaker, J. Lee, M. Mann, F. Merhout, A. Volfovsky, Exposure to opposing views on social media can increase political polarization. *Proc. Natl. Acad. Sci. U.S.A.* **115**, 9216–9221 (2018).

58. M. Mosleh, C. Martel, D. Eckles, D. G. Rand, Shared partisanship dramatically increases social tie formation in a twitter field experiment. *Proc. Natl. Acad. Sci. U.S.A* **118**, e2022761118 (2021).

59. M. W. Wagner, M. Gruszczynski, Who gets covered? Ideological extremity and news coverage of members of the U.S. congress, 1993 to 2013. *Journal. Mass Commun. Q.* **95**, 670–690 (2018).

60. S. Klar, Y. Krupnikov, J. B. Ryan, Affective polarization or partisan disdain?: Untangling a dislike for the opposing party from a dislike of partisanship. *Public Opin. Q.* **82**, 379–390 (2018).

61. S. Klar, Y. Krupnikov, *Independent Politics* (Cambridge Univ. Press, 2016).

62. C. Wells, L. A. Friedland, C. Hughes, D. V. Shah, J. Suk, M. W. Wagner, News media use, talk networks, and anti-elitism across geographic location: Evidence from wisconsin. *Int. J. Press/Politics* **26**, 438–463 (2021).

63. L. A. Friedland, D. V. Shah, M. W. Wagner, K. J. Cramer, C. Wells, J. Pevehouse, Battleground: Asymmetric communication ecologies and the erosion of civil society in Wisconsin, in *Elements in Politics and Communication* (2022).

64. N. P. Kalmoe, L. Mason, Lethal mass partisanship, p. 41.

65. S. C. McGregor, L. Molyneux, Twitter's influence on news judgment: An experiment among journalists. *Journalism* **21**, 597–613 (2020).

66. F. R. Baumgartner, B. D. Jones, *Agendas and Instability in American Politics* (University of Chicago Press, 2010).

67. Z. Terechshenko, F. Linder, V. Padmakumar, F. Liu, J. Nagler, J. A. Tucker, R. Bonneau, *A Comparison of Methods in Political Science Text Classification: Transfer Learning Language Models for Politics*, Presented at the XXXVII PolMeth Annual Meeting (2020).

68. T. Mikolov, K. Chen, G. Corrado, J. Dean, Efficient estimation of word representations in vector space, in *Proceedings of the 1st International Conference on Learning Representations, ICLR 2013* (Workshop Track Proceedings, Scottsdale, Arizona, USA, 2 to 4 May 2013).

69. D. P. Kingma, J. Ba, Adam: A method for stochastic optimization, in *Proceedings of the 3rd International Conference on Learning Representations, ICLR 2015* (Conference Track Proceedings, San Diego, CA, USA, 7 to 9 May 2015).

70. A. Russel, U.S. senators on twitter: Asymmetric party rhetoric in 140 characters. *Am. Politics Res.* **46**, 695–723 2018.

71. J. Zilinsky, C. Vaccari, J. Nagler, J. A. Tucker, Don’t Republicans tweet too? Using twitter to assess the consequences of political endorsements by celebrities. *Perspect. Polit.* **18**, 144–160 (2020).
